# Targeted LC–MS/MS Phytochemical Profiling and Synergistic Anti-Urease Activity of *Limoniastrum guyonianum* Extracts Supported by Molecular Docking and *In Silico* ADME and Drug-Likeness Assessment

**DOI:** 10.3390/molecules31203702

**Published:** 2026-10-15

**Authors:** Meryem Bouzid, Fouzia Berkani, Lamia Aouar, Ibtissem Sanah, Maria D’Elia, Rayana Boubekeur, Hichem Khammar, Chawki Bensouici, Mustafa Abdulah Yilmaz, Oguz Cakir, Abbas Tarhan, Amel Benbott, Luca Rastrelli

**Affiliations:** 1 Laboratory of Biomolecules and Plant Breeding, University of Oum El Bouaghi, BP 358, Oum El Bouaghi 04000, Algeria; fouzia.berkani@univ-oeb.dz (F.B.); aouar.lamia@univ-oeb.dz (L.A.); 2 Institute of Technology—Ain M’lila, University of Oum El Bouaghi, BP 358, Oum El Bouaghi 04000, Algeria; sanah.ibtissem@univ-oeb.dz; 3 Laboratoire de Recherche en Sciences Alimentaires, Formulation, Innovation, Valorisation et Intelligence Artificielle (SAFIVIA), Institut de la Nutrition, de l’Alimentation et des Technologies Agro-Alimentaires (INATAA), Université Frères Mentouri Constantine 1, Constantine 25000, Algeria; 4 Department of Pharmacy, University of Salerno, Via Giovanni Paolo II, 132, 84084 Fisciano, Italy; mdelia@unisa.it; 5 National Biodiversity Future Center—NBFC, 90133 Palermo, Italy; 6 Laboratory of Valorization and Promotion of Saharan Resources (VPRS), Faculty of Mathematics and Matter Sciences, University of Kasdi Merbah, Ouargla 30000, Algeria; boubekeur.rayana@univ-ouargla.dz; 7 Laboratory of Functional Ecology and Environment, University of Oum El Bouaghi, BP 358, Oum El Bouaghi 04000, Algeria; hichem.khammar@univ-oeb.dz; 8 Biotechnology Research Centre, Ali Mendjli New Town UV 03, BP E73, Constantine 25000, Algeria; chawkiislam@yahoo.fr; 9 Department of Analytical Chemistry, Faculty of Pharmacy, Dicle University, 21280 Diyarbakir, Turkey; mustafaabdullahyilmaz@gmail.com; 10 Science and Technology Application and Research Center, Dicle University, 21280 Diyarbakir, Turkey; abbas_tarhan@hotmail.com; 11 Department of Nutrition and Dietetics, Faculty of Health Sciences, Dicle University, 21280 Diyarbakir, Turkey; ocakir44@gmail.com; 12 Laboratory of Biological Engineering, Valorisation and Innovation of Agri-Food Products, ISTA Institute of Ain M’Lila, University of Oum El Bouaghi, BP 358, Oum El Bouaghi 04000, Algeria; malika1959@yahoo.fr

**Keywords:** *Limoniastrum guyonianum*, urease inhibition, synergistic activity, LC–MS/MS, phenolic compounds, antibacterial activity, molecular docking, ADME, drug likeness

## Abstract

*Limoniastrum guyonianum* Durieu ex Boiss. is an endemic halophyte native to the semi-arid regions of North Africa and represents an under-explored source of bioactive phytochemicals. This study investigated the phytochemical composition and anti-urease potential of *L. guyonianum* aerial parts through an integrated experimental and computational approach. Plant material was subjected to sequential organic extraction and an additional independent aqueous extract was prepared from the raw plant; the resulting organic fractions were characterized using a targeted LC–MS/MS method validated for 53 phytochemicals. Overall, 21 compounds, mainly flavonoids and phenolic acids, were quantified, 11 of which are quantitatively reported in this species for the first time. The hydromethanolic and methanolic extracts showed the greatest phytochemical diversity, containing 17 and 16 compounds, respectively, together with high total phenolic contents (222.39 ± 0.61 and 157.65 ± 4.40 µg GAE/mg) and flavonoid contents (114.34 ± 16.82 and 223.62 ± 10.64 µg QE/mg). The anti-urease activity of *L. guyonianum* was evaluated for the first time; the combination of polar extracts showed substantial inhibitory activity (IC_50_ = 44.66 ± 1.21 µg/mL) compared with the reference inhibitor thiourea (IC_50_ = 19.62 ± 0.34 µg/mL). Combination analysis yielded CI point estimations below 1 across the non-extrapolated effect range (CI = 0.52–0.69 at Fa = 0.10–0.40), together with substantial dose-reduction effects (DRI ≥ 8.24 for ME and ≥1.75 for MEW). The hydromethanolic extract also exhibited preliminary antibacterial activity against *Bacillus cereus* and *Staphylococcus aureus*, with inhibition zones of 18.50 ± 0.07 and 19.50 ± 0.07 mm, respectively. Molecular docking against three urease structures (PDB IDs: 1E9Y, 4H9M, and 4UBP) identified epigallocatechin as the highest-ranked phytochemical according to the composite CNN_VS metric (3.80 ± 0.31), with an average predicted binding energy of −7.55 kcal·mol^−1^. *In silico* ADME and drug-likeness assessment further revealed marked differences in predicted oral pharmacokinetic properties among the quantified phytochemicals, with lower-molecular-weight aglycones generally showing more favorable profiles than glycosylated and highly hydroxylated compounds. These findings provide the first evidence of the anti-urease potential of *L. guyonianum* and highlight the synergistic activity of its polar extracts, supporting further investigation of this North African halophyte as a source of bioactive phytochemicals.

## 1. Introduction

Urease is a nickel-dependent metalloenzyme widely distributed among bacteria, archaea, and plants that catalyze the hydrolysis of urea into ammonia and carbon dioxide. The resulting increase in local pH can promote bacterial survival and colonization and contribute to the pathogenesis of several urease-associated disorders, including gastritis, peptic ulcer disease, urinary tract infections, and nephrolithiasis [1,2]. Therefore, urease represents an important pharmacological target, particularly in infections caused by urease-producing micro-organisms. Moreover, the increasing prevalence of antimicrobial resistance is progressively compromising the effectiveness of conventional antibiotics and imposing substantial clinical and economic burdens worldwide [3,4], highlighting the need for alternative or complementary antimicrobial strategies [5]. In this context, bioactive compounds capable of simultaneously interfering with bacterial virulence mechanisms, such as urease activity, and microbial growth are of particular interest [2,6,7]. Although synthetic urease inhibitors, including acetohydroxamic acid and thiourea derivatives, have been extensively investigated, their application may be limited due to toxicity, chemical instability, and unfavorable pharmacokinetic properties [8]. Consequently, natural products have attracted increasing attention as potential sources of novel urease inhibitors [9,10]. The concomitant inhibition of urease activity and bacterial proliferation may provide a complementary strategy targeting both enzymatic virulence factors and microbial viability [11,12,13,14].

Medicinal plants remain an important source of structural diverse bioactive compounds and continue to contribute to the discovery of pharmacologically active molecules [15,16]. In this respect, North African flora is particularly interesting because species adapted to arid, saline, and Mediterranean environments accumulate frequently phenolic compounds and other stress-responsive secondary metabolites. In Algeria, investigations specifically addressing anti-urease activity remain comparatively limited. A butanolic extract of Algerian *Crataegus monogyna*, for example, showed marked urease inhibition (IC_50_ = 26.36 µg/mL), supporting the potential contribution of its phenolic constituents to this activity [17]. Similarly, Algerian prickly pear (*Opuntia ficus-indica*) seed by-products have been evaluated for their inhibitory effects against urease and other enzymes, illustrating the potential of plant-derived residues as sources of multifunctional bioactive compounds [18]. More recently, methanolic leaf and rhizome extracts of the eastern Algerian seagrass *Posidonia oceanica* exhibited substantial urease inhibition, with IC_50_ values of 15.78 and 20.52 µg/mL, respectively; also, LC–MS/MS profiling and molecular docking suggested a possible contribution of phenolic constituents to the observed activity [19].

Nevertheless, large portions of North Africa endemic and halophytic flora remain pharmacologically underexplored. In southeastern Algeria, exposure to extreme aridity and salinity promotes the accumulation of secondary metabolites involved in plant adaptation to environmental stress [20]. Among these species, *Limoniastrum guyonianum* Durieu ex Boiss. is a halophytic plant adapted to harsh environmental conditions and represents a promising, but still poorly investigated, source of bioactive phytochemicals. Its tolerance to abiotic stress has been associated with a phytochemical profile rich in phenolic compounds potentially responsible for several biological activities [21,22]. Although previous studies have reported antioxidant and antimicrobial properties of *L. guyonianum* extracts [23,24,25,26,27], its quantitative phytochemical composition and pharmacological potential, particularly its anti-urease activity, remain insufficiently characterized. This represents an important knowledge gap, since polyphenols and flavonoids can inhibit urease through different mechanisms, including interactions with catalytic site, coordination with dinickel center, and modulation of enzyme conformation [28]. Moreover, phenolic compounds frequently display multiple biological activities, including both urease inhibition and antimicrobial effects [29], making phenolic-rich extracts particularly relevant for multifunctional bioactivity research.

Accordingly, the present study aimed to characterize the phytochemical composition of *L. guyonianum* aerial-part extracts obtained using solvents of increasing polarity through targeted LC–MS/MS profiling and investigate their anti-urease and antibacterial activities. Particular attention was given to the interaction between methanolic and hydromethanolic extracts, with combination effects quantitatively assessed using the Chou–Talalay approach. Molecular docking against bacterial and plant ureases was subsequently used to investigate the potential interactions of the quantified phytochemicals with the urease catalytic site, while SwissADME (accessed on 25 August 2026) was used to assess their predicted physicochemical, pharmacokinetic, and drug-likeness properties. This study provides the first evidence of the anti-urease activity of *L. guyonianum* and explores the contribution of its phenolic constituents to the observed biological effects, through integrating phytochemical characterization, experimental bioactivity assessment, combination analysis, and computational approaches.

## 2. Results 

### 2.1. Extraction Yields

Extraction yield varied markedly according to the extraction solvent (Table 1). The aqueous extract, which was prepared separately, showed the highest overall yield (16.35%). Among the sequential solvent extracts, the hydromethanolic extract (MEW), provided the highest yield (4.16%), followed by the methanolic extract (ME; 3.74%), petroleum ether extract (PE; 1.21%), and dichloromethane extract (DCM; 0.67%).

A significant effect of extraction solvent on percentage yield was observed by one-way ANOVA (F(4, 10) = 7606.65, *p* < 0.001). Tukey’s HSD post hoc test showed significant differences among all five extracts (*p* < 0.05), as reported in Table 1.

### 2.2. Total Phenolic, Flavonoid, and Condensed Tannin Contents

The distribution of phenolic compounds, flavonoids, and condensed tannins varied substantially among the analyzed extracts (Table 2). Hydromethanolic extract (MEW) showed the highest total phenolic content (TPC), reaching 222.39 µg GAE/mg, then the methanolic extract (ME; 157.65 µg GAE/mg). Lower TPC values were recorded for dichloromethane (DCM; 57.46 µg GAE/mg) and petroleum ether (PE; 43.51 µg GAE/mg) extracts.

A different distribution was observed for total flavonoid content (TFC). The methanolic extract exhibited the highest value (223.62 µg QE/mg), followed by the hydromethanolic extract (114.34 µg QE/mg), DCM extract (85.67 µg QE/mg), and PE extract (23.67 µg QE/mg).

Condensed tannin content (CTC) was also highest in the methanolic extract (29.83 µg CE/mg), closely followed by the hydromethanolic extract (27.80 µg CE/mg). Lower concentrations were detected in DCM (16.67 µg CE/mg) and PE (10.57 µg CE/mg) extracts.

Overall, the quantitative data showed that ME and MEW, contained the highest levels of the measured phenolic-related constituents, although the relative distribution differed among TPC, TFC, and CTC.

### 2.3. Targeted LC–MS/MS Metabolite Profiling

**Figure 1:**
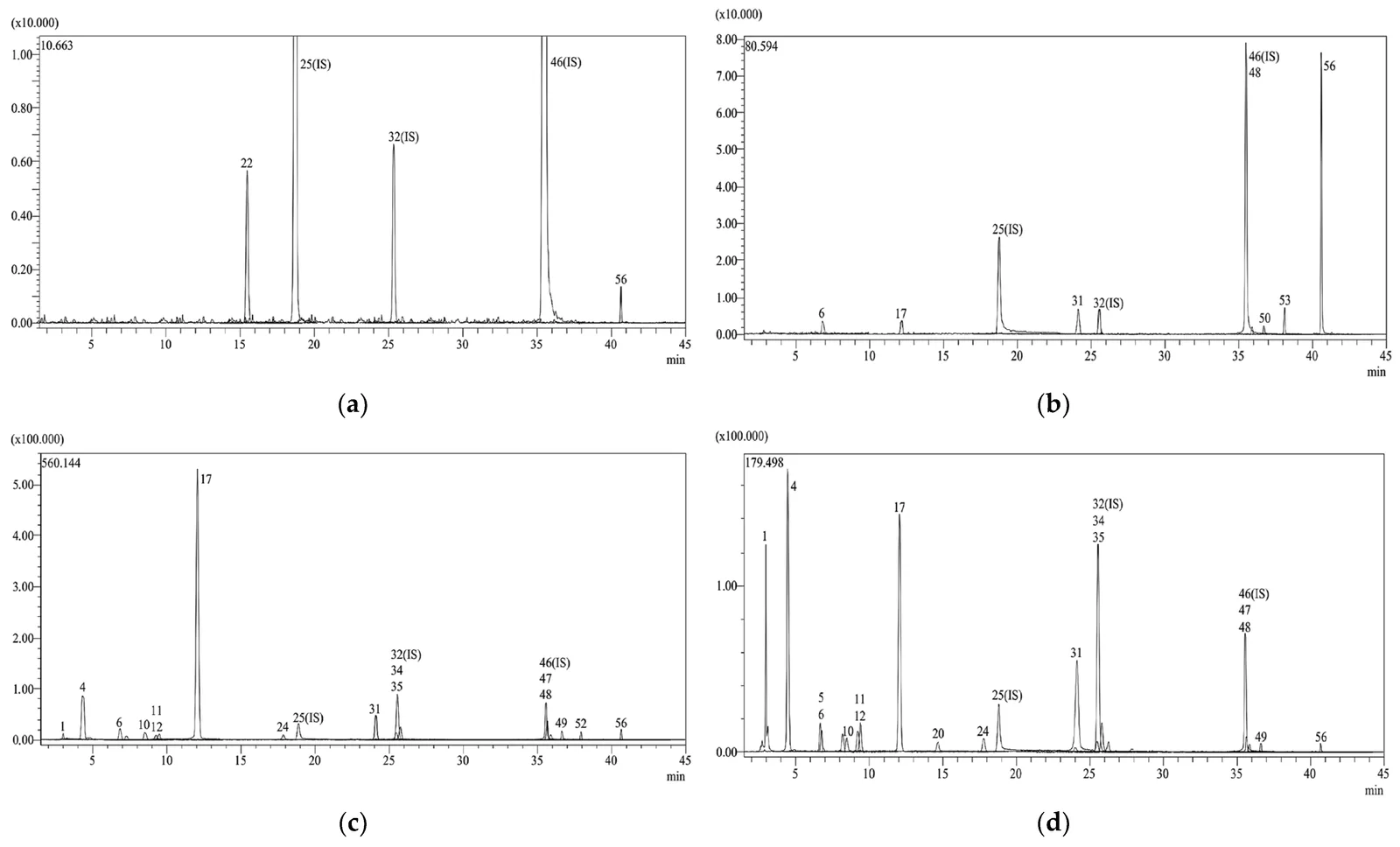
Representative LC–MS/MS chromatograms of *Limoniastrum guyonianum* extracts: (**a**) petroleum ether (PE), (**b**) dichloromethane (DCM), (**c**) methanol (ME), and (**d**) methanol–water (MEW; 5:1, *v/v*) extracts. Peaks labeled IS indicate the internal standards.

Targeted LC–MS/MS analysis was performed using a 53-analyte phytochemical panel. Overall, 21 compounds were quantified above the established limit of quantification (LOQ) in at least one *L. guyonianum* extract (Table 3), whereas the remaining analytes were not quantitatively detected under the applied analytical conditions. Representative chromatograms of PE, DCM, ME, and MEW extracts are shown in Figure 1; the aqueous extract was not subjected to LC–MS/MS analysis. Complete validation data for the 53-analyte method are provided in Supplementary Table S1.

The phytochemical profiles differed markedly among the four sequential extracts. The methanolic and hydromethanolic fractions showed the greatest phytochemical diversity, with 16 and 17 quantified compounds, respectively, whereas substantially fewer metabolites were detected in PE and DCM fractions. Flavonoids represented the predominant chemical class, accounting for 13 of the 21 quantified metabolites.

Miquelianin was the most abundant quantified compound, reaching 5333 µg/g and 4054 µg/g in MEW and ME extracts, respectively. Gallic acid was also present at high concentrations in both polar extracts, with values of 4518 µg/g in MEW and 3396 µg/g in ME. Epigallocatechin was detected exclusively in the MEW fraction at 4267 µg/g.

Other abundant constituents of the methanolic extract included caffeic acid (3525 µg/g) and isoquercitrin (1733 µg/g), whereas MEW extract contained substantial amounts of epigallocatechin gallate (1333 µg/g), quinic acid (1322 µg/g), and isoquercitrin (1000 µg/g). Several compounds showed a more restricted distribution among evaluated fractions. Epicatechin gallate was quantified only in PE extract (53 µg/g), whereas syringic aldehyde was detected only in MEW extract (35.0 µg/g). Luteolin and apigenin were detected only in DCM fraction, while kaempferol was detected only in ME extract.

Overall, the LC–MS/MS data revealed a clear concentration of quantified phytochemicals in ME and MEW extracts, both in terms of numbers of detected metabolites and the abundance of several major constituents.

### 2.4. Urease Inhibitory Activity

The urease inhibitory activity of the *L. guyonianum* extracts differed markedly according to the extraction fraction (Table 4). PE, DCM, and AQ extracts did not reach 50% inhibition within the experimentally tested concentration range (3.125–200 µg/mL). Also, the ME extract did not reach its modeled IC_50_ within the experimental concentration range; although inhibition percentage at the highest tested concentration was 44% for the ME extract. In contrast, MEW extract showed substantially greater activity, with an IC_50_ of 54.43 µg/mL. The fixed 1:1 combination of ME and MEW produced the strongest inhibitory effect among the tested extract preparations, with an IC_50_ value of 44.66 µg/mL. The reference inhibitor thiourea remained more potent, with an IC_50_ value of 19.62 µg/mL.

To avoid interpretation based on doses extrapolated beyond the experimentally tested concentration range, combination analysis was primarily evaluated over Fa = 0.10–0.40. CI point estimates remained below 1 throughout this non-extrapolated range (CI = 0.518–0.689), while DRI values were ≥8.24 for ME and ≥1.75 for MEW, indicating a substantial dose-reduction effect for both components when combined. The complete Fa-CI-DRI analysis and bootstrap confidence intervals are provided in Supplementary Table S2. Dose-response curves showed satisfactory model fitting for ME, MEW, combined ME-MEW, and thiourea, with *R*^2^ values of 0.9914, 0.9580, 0.9956, and 0.9976, respectively.

### 2.5. Antibacterial Activity

The antibacterial activity of *L. guyonianum* extracts varied according to both extract type and bacterial strain (Table 5). Among the tested fractions, MEW extract showed the most preliminary antibacterial activity against the Gram-positive strains. Against *Bacillus cereus*, MEW produced an inhibition zone of 18.50 mm at the highest tested concentration, whereas the aqueous extract showed a smaller inhibition zone of 7.00 mm. PE, DCM, and ME extracts showed weak or no detectable inhibition under the same experimental conditions. For *Staphylococcus aureus*, MEW produced the largest inhibition zone, reaching 19.50 mm at the highest tested concentration. Also, ME extract showed marked preliminary activity, with an inhibition zone of 18.50 mm, while DCM extract reached 15.50 mm. Translucent zones were additionally observed for the methanolic and hydromethanolic extracts at the highest tested concentration. Activity against the Gram-negative strains was generally lower and more variable. Against *Escherichia coli*, the methanolic extract produced the largest inhibition zone among the plant extracts (8.00 mm), whereas the remaining fractions showed weak or no detectable inhibition at most tested concentrations. Against *Salmonella enteritidis*, the hydromethanolic extract showed the greatest inhibition among the tested plant fractions, reaching 16.50 mm, followed by the methanolic extract at 14.00 mm. Lower activity was observed for the remaining extracts. Overall, the antibacterial screening showed a stronger response of the Gram-positive strains, particularly *S. aureus* and *B. cereus*, to the polar extracts, with MEW displaying the highest inhibition zones among the tested *L. guyonianum* fractions.

### 2.6. Molecular Docking and Target Validation

Twenty phytochemicals quantified in *L. guyonianum* were subjected to molecular docking against three crystallographically characterized ureases: *Helicobacter pylori* urease (PDB ID: 1E9Y), jack bean (*Canavalia ensiformis*) urease (PDB ID: 4H9M), and *Sporosarcina pasteurii* urease (PDB ID: 4UBP). Acetohydroxamic acid (AHA) was included as the reference urease inhibitor. Twenty independent docking runs with different seeds were performed for each ligand–target pair, generating 1200 retained phytochemical poses which yielded 8268 protein–ligand contact records; when combined with the 144 interaction records obtained from a subset of the AHA redocking poses used for validation, this resulted in a total of 8412 analyzed contact records. The successful redocking of AHA supported the suitability of the three receptor structures for subsequent analysis. CNNscore values of 0.933, 0.906, and 0.940 were obtained for 1E9Y, 4H9M, and 4UBP, respectively, all exceeding the predefined validation threshold of 0.90. Corresponding RMSD values were also below the predefined 2.0 Å threshold (Table 6).

The three receptor models exhibited distinct docking profiles as reflected in their mean CNN scores; 1E9Y and 4UBP yielded the highest values (0.66 and 0.65, respectively), whereas 4H9M showed a lower mean score of 0.55. The median predicted binding energies also varied across targets, with 4UBP exhibiting the most favorable predicted affinity at −7.23 kcal⋅mol^−1^. Furthermore, the frequency of poses coordinating with the catalytic Ni^2+^ ions was receptor-dependent, ranging from a high of 86.4% in 1E9Y to 63.9% in 4UBP (Table 6).

#### 2.6.1. Compound Ranking and Cross-Target Scoring

The 20 phytochemicals displayed distinct ranking profiles according to CNN_VS and Vina-derived binding energy (Table 7). Among the natural compounds, epigallocatechin showed the highest mean CNN_VS value (3.80), followed by epicatechin gallate (3.61), gallic acid (3.52), and epigallocatechin gallate (3.44). AHA showed a higher CNN_VS value than all tested phytochemicals (4.67). Ranking based on Vina-derived binding energy differed substantially from CNN_VS ranking. Hesperidin exhibited the most favorable mean binding energy prediction among the phytochemicals (−9.62 kcal·mol^−1^), followed by epigallocatechin gallate (−9.08 kcal·mol^−1^), epicatechin gallate (−8.89 kcal·mol^−1^), miquelianin (−8.83 kcal·mol^−1^), and quercetin (−8.82 kcal·mol^−1^). In contrast, AHA showed a less negative mean Vina-derived binding energy (−4.25 kcal·mol^−1^) despite its higher CNN_VS score. In the present study, the application of the predefined pK_calc threshold of ≥6.30 identified six phytochemicals as potentially active candidates: epicatechin gallate, epigallocatechin gallate, quercetin, isoquercitrin, hesperidin, and miquelianin. Five of these six compounds were quantified in the ME and MEW extracts, which were also the fractions showing the greatest experimental urease inhibition (Table 7). Cross-target analysis showed consistently strong correlations for Vina-derived binding energy among the three urease structures, with Spearman’s ρ values ranging from 0.901 to 0.916 (Table 8). CNN_VS showed greater target dependence, with correlations ranging from ρ = 0.347 to 0.783; statistically, the correlation between 1E9Y and 4H9M was not significant (Table 8).

#### 2.6.2. Protein–Ligand Interaction Profiles

**Figure 2:**
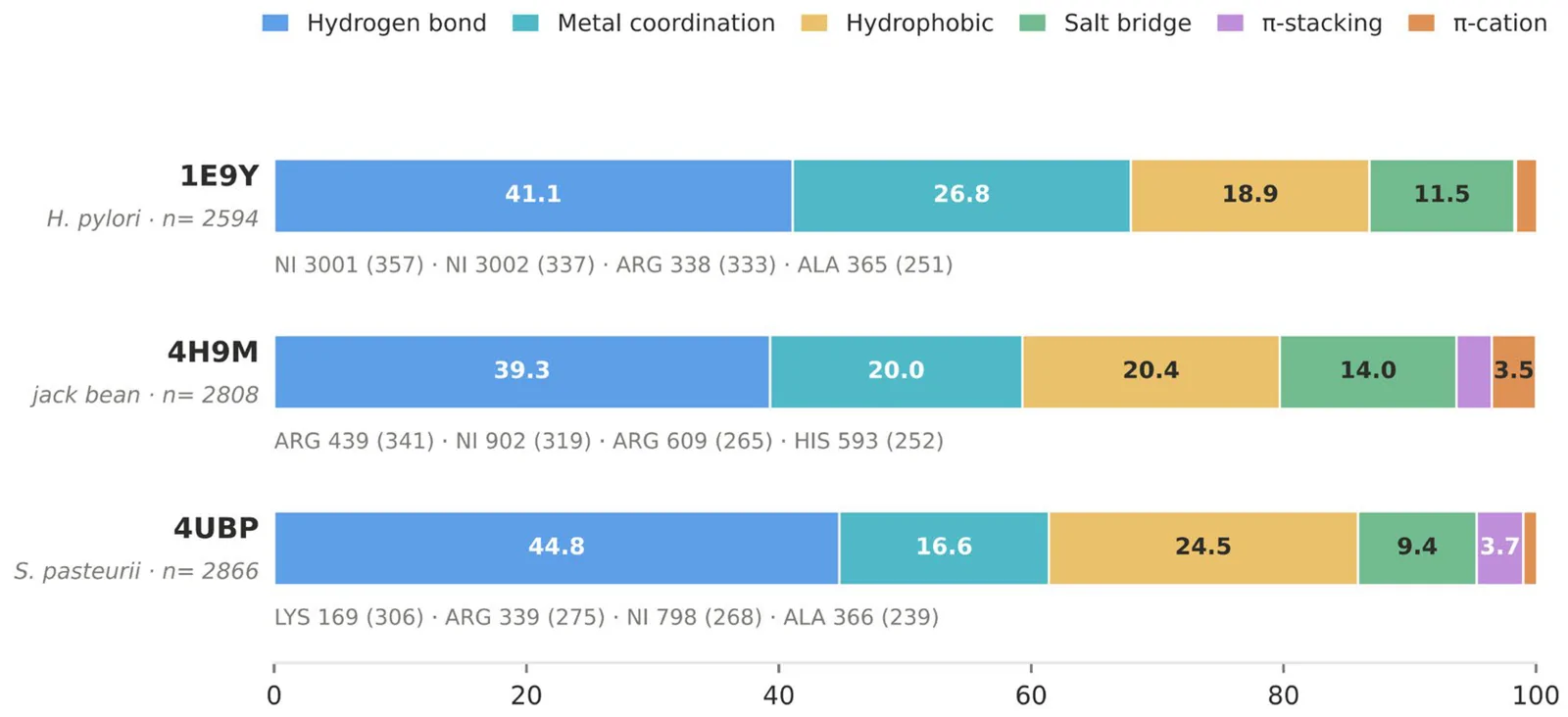
Distribution of predicted protein–ligand interaction classes across the three urease receptors for the 20 phytochemicals. Stacked bars show the percentage contribution of hydrogen bonds, metal coordination, hydrophobic contacts, salt bridges, π stacking, and π–cation interactions to all contacts detected for each receptor (1E9Y, 2594 contacts; 4H9M, 2808 contacts; 4UBP, 2866 contacts; total = 8268 contacts). The four most frequently contacted residues or catalytic metal sites are annotated below each bar. Contact counts were obtained from PLIP analysis of the 1200 retained phytochemical poses (20 compounds × 20 independent runs × 3 receptors); AHA validation poses were excluded.

Analysis of the protein–ligand contact dataset revealed target-dependent differences in the relative contribution of the major interaction classes. Hydrogen bonding was the predominant interaction type across all three urease structures, representing approximately 39.3–44.8% of the detected interactions. Metal coordination represented 26.8%, 20.0%, and 16.6% in the 1E9Y, 4H9M, and 4UBP interactions, respectively. Hydrophobic interactions increased across the three receptor models. Salt bridges represented approximately 9.4–14.0% of the detected contacts. Aromatic interactions were less frequent and showed greater target dependence, with π stacking ranging from approximately 0.1% in 1E9Y to 3.7% in 4UBP. Residue-level analysis further revealed distinct interaction patterns among the three receptors. In 1E9Y, the two catalytic nickel ions (Ni3001 and Ni3002) were among the most frequently contacted sites, with 357 and 337 contacts, respectively. Arg338 and Ala365 were also frequently involved in ligand interactions. In 4H9M, Arg439 was the most frequently contacted residue (341 contacts), whereas in 4UBP, the interaction pattern shifted toward peripheral residues, particularly Lys169 (306 contacts) and Arg339 (275 contacts) (Figure 2). The four highest-ranked phytochemicals according to CNN_VS—epigallocatechin, epicatechin gallate, gallic acid, and epigallocatechin gallate—expressed frequent interactions with the dinickel catalytic center. Ni^2+^-coordination frequencies were 83.3%, 86.7%, 100.0%, and 86.7%, respectively. In contrast, hesperidin, despite showing the most favorable Vina-derived binding energy, ranked 14th according to CNN_VS and expressed a different interaction profile characterized by a larger number of protein contacts and a lower relative contribution of direct metal coordination.

#### 2.6.3. Binding Modes of Selected Phytochemicals

Detailed analysis of the selected poses for the four highest-ranked CNN_VS phytochemicals and hesperidin across the three receptors yielded 15 representative ligand–receptor complexes (Table 9 and Table 10). Collectively, these poses represented 132 detected protein–ligand interactions, including 58 hydrogen bonds, 21 hydrophobic contacts, 25 Ni^2+^ coordination contacts, 15 salt bridges, eight π–cation interactions, and five π-stacking interactions.

Hydrogen-bond distances ranged from 1.81 to 3.55 Å, hydrophobic contacts from 3.21 to 3.98 Å, and ligand–Ni^2+^ distances from 1.79 to 3.38 Å. 14 of the 15 selected poses contained at least one ligand donor atom within 3.5 Å of a catalytic nickel ion, indicating frequent direct interaction with the dinickel center. Also, 14 poses interacted with residues belonging to the mobile-flap region and with the conserved flap arginine.

**Figure 3:**
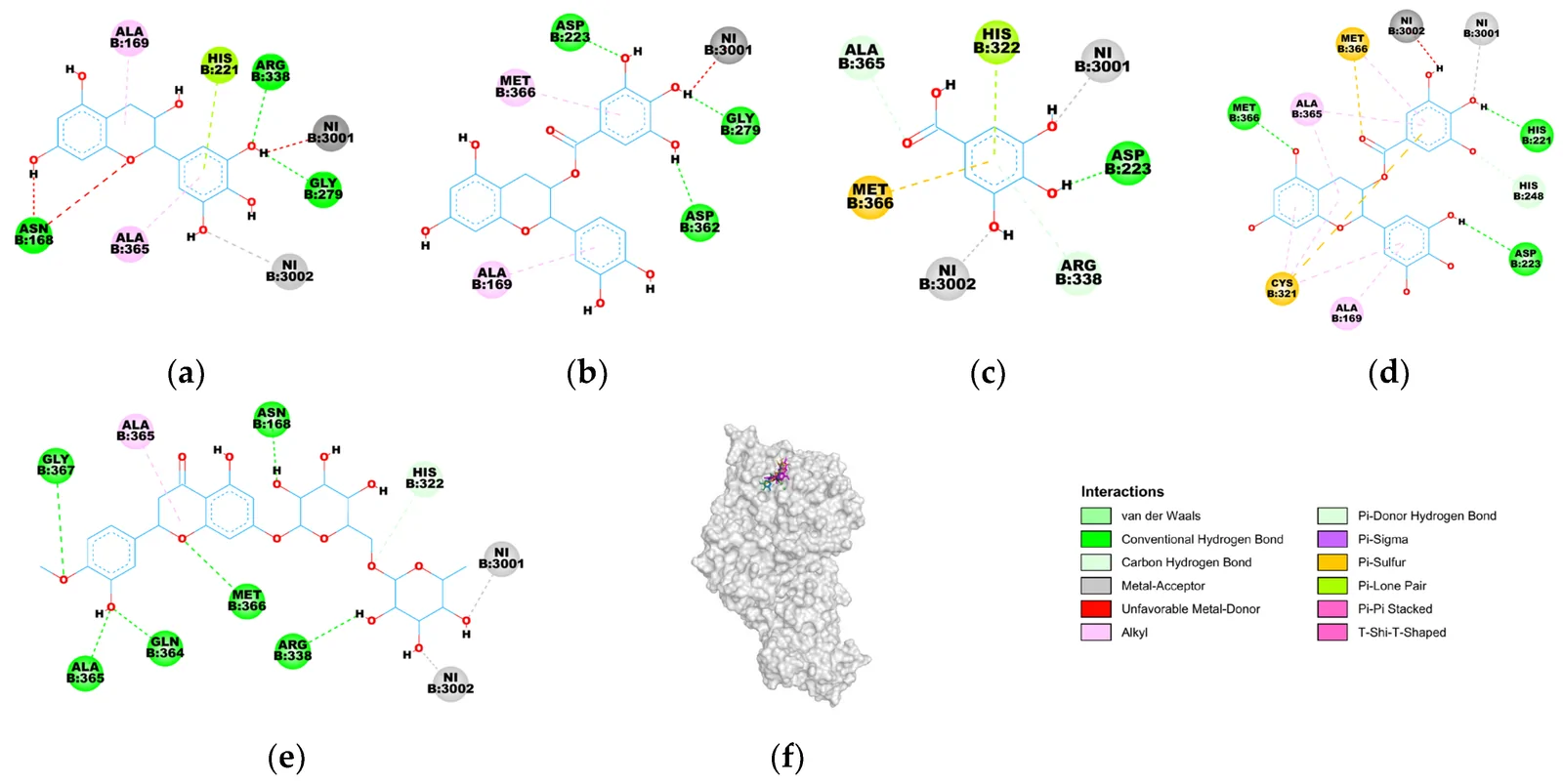
Representative binding modes of selected phytochemicals in *Helicobacter pylori* urease (PDB ID: 1E9Y). Two-dimensional protein–ligand interaction diagrams are shown for (**a**) epigallocatechin, (**b**) epicatechin gallate, (**c**) gallic acid, (**d**) epigallocatechin gallate, and (**e**) hesperidin. (**f**) Three-dimensional surface representation of the 1E9Y urease showing the location of the selected ligands within the binding pocket. Interaction types are indicated according to the color legend.

**Figure 4:**
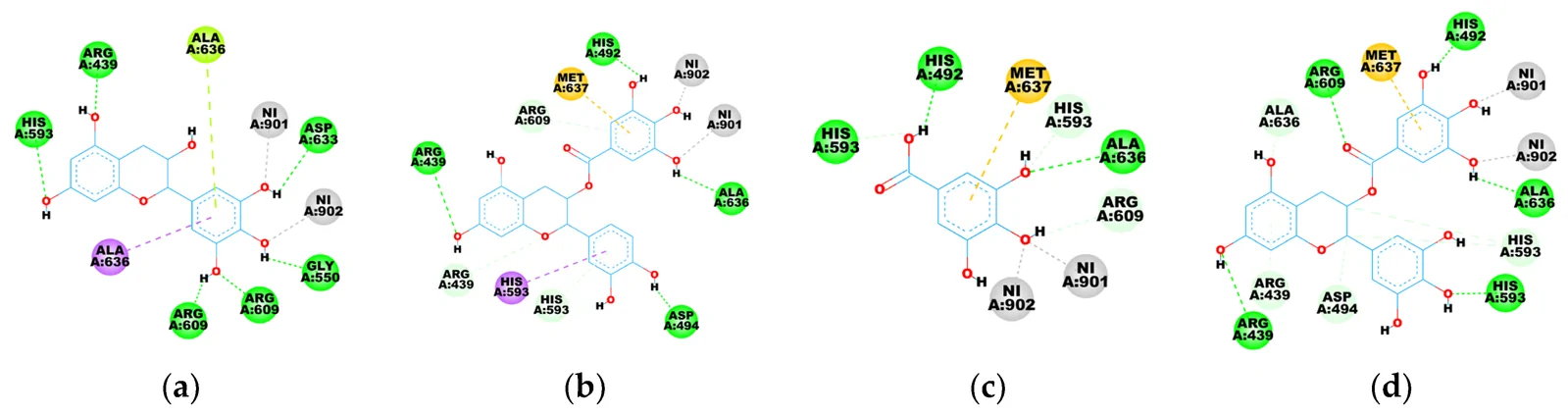

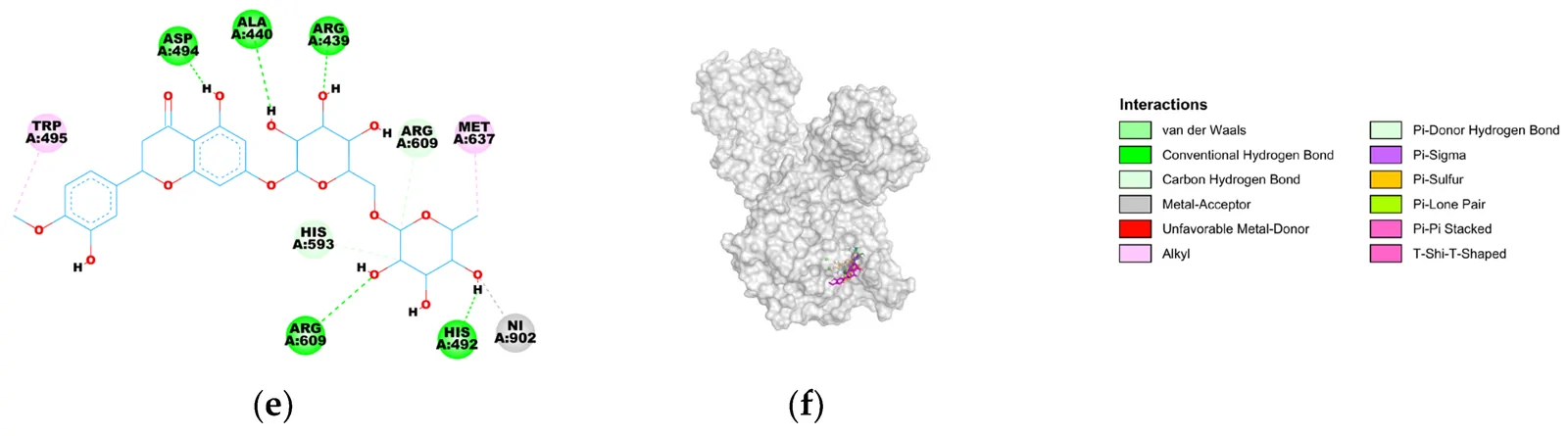
Representative binding modes of selected phytochemicals in jack bean urease (*Canavalia ensiformis*; PDB ID: 4H9M). Two-dimensional protein–ligand interaction diagrams are shown for (**a**) epigallocatechin, (**b**) epicatechin gallate, (**c**) gallic acid, (**d**) epigallocatechin gallate, and (**e**) hesperidin. (**f**) Three-dimensional surface representation of the 4H9M urease showing the location of the selected ligand within the binding pocket. Interaction types are indicated according to the color legend.

**Figure 5:**
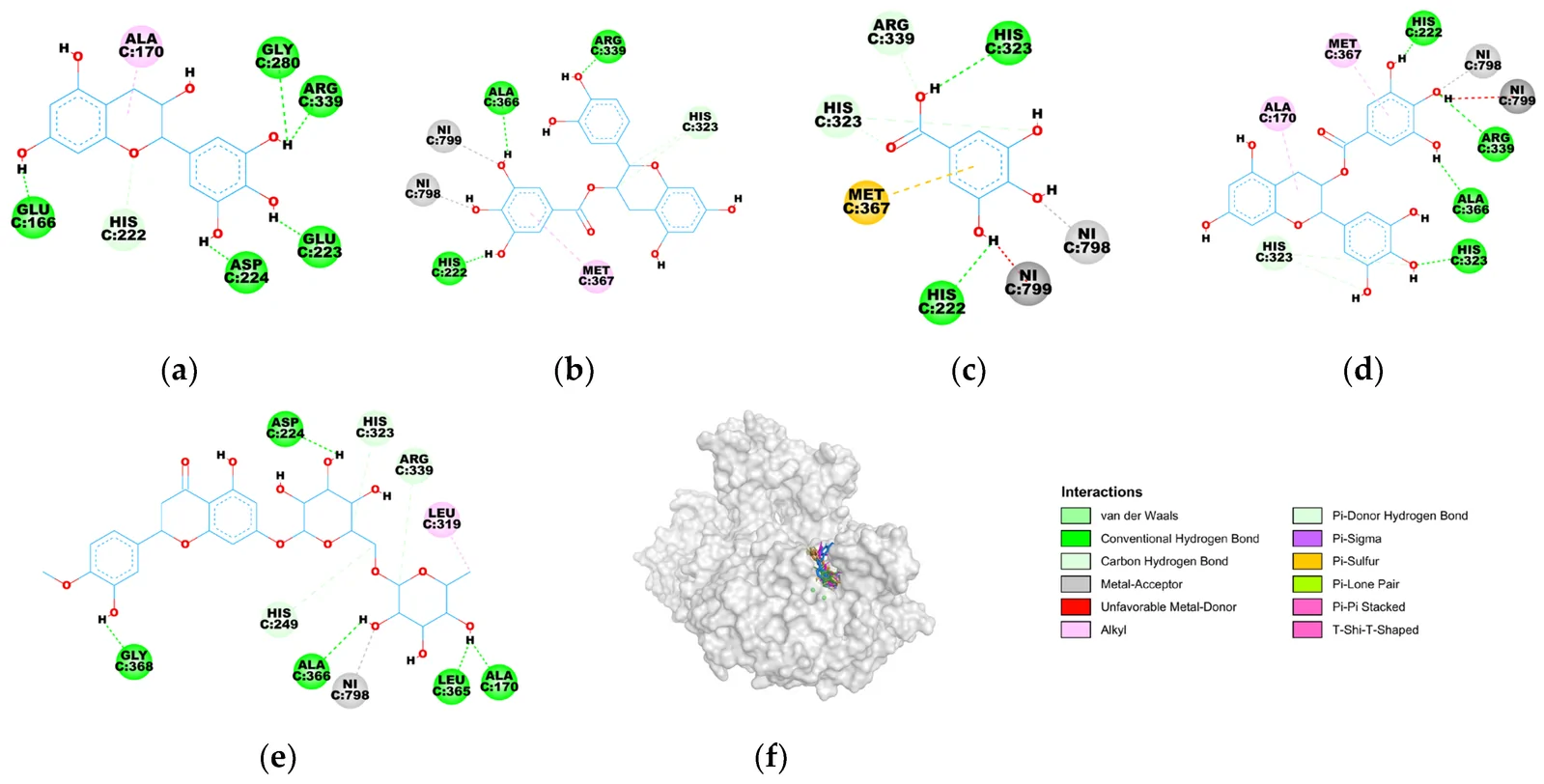
Representative binding modes of selected phytochemicals in *Sporosarcina pasteurii* urease (PDB ID: 4UBP). Two-dimensional protein–ligand interaction diagrams are shown for (**a**) epigallocatechin, (**b**) epicatechin gallate, (**c**) gallic acid, (**d**) epigallocatechin gallate, and (**e**) hesperidin. (**f**) Three-dimensional surface representation of the 4UBP urease showing the location of the selected ligands within the binding pocket. Interaction types are indicated according to the color legend.

Among the selected phytochemicals, gallic acid showed particularly consistent metal coordination, interacting with both catalytic Ni^2+^ ions in the representative poses obtained. all three receptors. The closest ligand–metal distances were 2.03 Å, 2.64 Å, and 2.17 Å for 1E9Y, 4H9M, and 4UBP respectively. (Figure 3, Figure 4 and Figure 5) By contrast, hesperidin displayed the highest number of hydrogen-bonding interactions among the selected compounds, with 16 hydrogen bonds across its three representative receptor complexes, but it showed less extensive direct involvement of the dinickel center.

Therefore, the interaction patterns differed substantially among compounds that ranked highly according to CNN_VS and those favored primarily by Vina-derived binding energy prediction, providing complementary structural information on their predicted binding behavior across the three urease targets.

### 2.7. In Silico ADME and Drug-likeness Profiles

SwissADME analysis revealed marked differences in predicted physicochemical and pharmacokinetic properties among the phytochemicals quantified in *L. guyonianum* (Table 11). The complete compound-level dataset is provided in Supplementary Table S4.

Generally, lower-molecular-weight phenolic derivatives and flavonoid aglycones displayed more favorable predicted oral drug-likeness profiles than the larger glycosylated and highly polyhydroxylated compounds. Generally, phenolic aldehydes, simple phenolic acids, flavones, flavanones, and flavonol aglycones showed low numbers of Lipinski Rule-of-Five violations, moderate lipophilicity, and comparatively favorable predicted gastrointestinal absorption.

Among flavonoid aglycones, the hesperetin, naringenin, quercetin, luteolin, acacetin, apigenin, and kaempferol showed physicochemical profiles compatible with conventional small-molecule drug-likeness criteria. Their predicted gastrointestinal absorption was generally higher than that of the corresponding glycosylated flavonoids.

**Figure 6:**
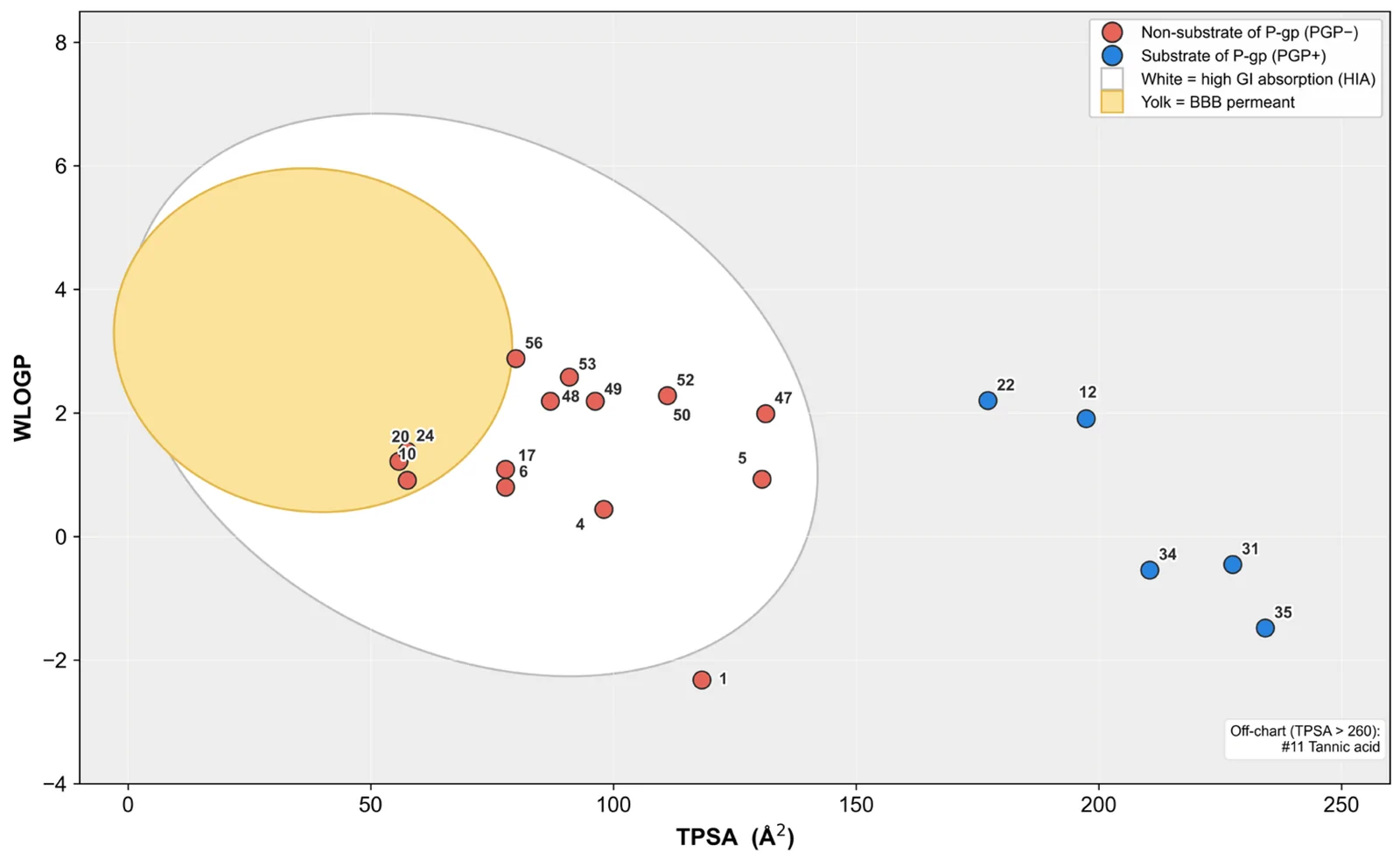
BOILED-Egg plot illustrating the predicted gastrointestinal absorption (white region) and blood–brain barrier (BBB) permeation (yellow yolk) of phytochemicals from *L. guyonianum* using SwissADME. The plot maps topological polar surface area (TPSA, Å^2^) against WLOGP. Data points are color-coded by P-glycoprotein (P-gp) substrate status; blue represents substrates (PGP+) and red represents non-substrates (PGP−).

By contrast, the flavonoid glycosides miquelianin, isoquercitrin, and hesperidin showed substantially higher molecular weights and topological polar surface area (TPSA), together with lower predicted oral bioavailability and reduced gastrointestinal absorption. Several of these compounds were also predicted to behave as P-glycoprotein substrates. Additionally, the BOILED-Egg analysis showed that these highly polar glycosylated structures were located outside the region associated with predicted efficient passive gastrointestinal absorption, whereas selected lower-molecular-weight compounds, specifically the seven above-named flavonoid aglycones, showed more favorable predicted permeability characteristics compared to their glycosylated counterparts (Figure 6).

Also, galloylated catechins displayed less favorable predicted oral pharmacokinetic properties than the lower-molecular-weight phenolic constituents. Epicatechin gallate and epigallocatechin gallate showed relatively high TPSA values and reduced passive permeability, despite their favorable docking performance against urease.

Quinic acid represented a distinct physicochemical profile among the lower-molecular-weight constituents. Despite its relative low molecular weight, its high polarity was associated with low predicted gastrointestinal absorption.

Overall, the SwissADME results showed an inverse trend between molecular complexity/polarity and predicted passive oral absorption. Generally, lower-molecular-weight aglycones and simple phenolic derivatives exhibited more favorable predicted drug-likeness properties. Glycosylation and extensive hydroxylation are associated with reduced predicted gastrointestinal permeability and oral bioavailability.

## 3. Discussion

The present study provides an integrated characterization of *Limoniastrum guyonianum* combining polarity-guided extraction, targeted LC–MS/MS profiling, urease inhibition, antibacterial screening, molecular docking, and *in silico* pharmacokinetic assessment. *L. guyonianum* is adapted to saline and arid environments in North Africa, where exposure to drought, salinity, and high temperatures can promote the accumulation of phenolic acids, flavonoids, tannins, and other stress-responsive secondary metabolites [31,32]. Despite this phytochemical potential, previous investigations of this species have mainly focused on crude extracts and general antioxidant, anti-inflammatory, cytotoxic, or antimicrobial activities [25,26,33], whereas its quantitative metabolite profile and anti-urease potential have remained comparatively poorly characterized. The marked dependence of extraction yield and chemical composition on solvent polarity observed here is consistent with previous evidence showing that *L. guyonianum* is particularly enriched in polar phenolic constituents [34]. Also, the sequential extraction approach has enabled the recovery of compounds to span a relatively broad physicochemical range, which is relevant because both antimicrobial and urease-inhibitory constituents may differ substantially in polarity and chemical behavior [10,35,36,37].

Targeted LC–MS/MS profiling confirmed that ME and MEW extracts were highly enriched in phenolic constituents. Specifically, miquelianin, gallic acid, caffeic acid, epigallocatechin, isoquercitrin, and epigallocatechin gallate were reported as the most abundant compounds within these polar fractions. Several of these metabolites or related phenolic constituents have previously been reported in *L. guyonianum* using RP-HPLC or centrifugal partition chromatography [21,25,33]. In contrast, as far as can be ascertained, quinic acid, tannic acid, protocatechuic aldehyde, syringic aldehyde, epicatechin gallate, isoquercitrin, hesperidin, naringenin, hesperetin, luteolin, and acacetin have not previously been quantitatively reported in this species. Previous studies have also highlighted chlorogenic acid as a main component of *L. guyonianum* [26]; the present detection of free quinic acid adds further evidence of the diversity of hydroxycinnamate-related metabolism in this species. Overall, the LC–MS/MS data substantially extend the quantitative phytochemical information currently available for *L. guyonianum*.

The concentration of phenolic constituents in the polar fractions was paralleled by their greater urease-inhibitory activity. MEW extract was substantially more active than ME, DCM, PE and AQ extracts, whereas the 1:1 ME–MEW combination produced the strongest inhibition among the tested extract preparations. Although this activity remained lower than that of thiourea, the observed IC_50_ of the combined extract is notable for an unpurified botanical preparation. Comparable plant-derived urease-inhibitory activities have previously been reported for phenolic-rich extracts [9]. In particular, ME extracts of *Posidonia oceanica* tested against jack bean urease using a similar indophenol-based assay showed IC_50_ values of 15.78 and 20.52 µg/mL for leaf and rhizome extracts, respectively [19,38]. Direct comparison among plant extracts should nevertheless be made cautiously because extraction procedures, phytochemical composition, assay conditions, and reference-inhibitor performance can substantially affect apparent potency.

An important finding of the present study was the synergistic interaction between ME and MEW fractions with 1:1 ratio. CI point estimates remained below 1 across the non-extrapolated effect level (Fa) of 0.20 onwards, while DRI values greater than 1 indicated dose-sparing effects for both components. This suggests that the observed activity is not attributable to any single constituent in isolation but rather, it arises from the complex interplay of multiple metabolites within the extract matrix. Polyphenols can interact with urease through multiple mechanisms, including coordination of the dinickel catalytic center, hydrogen bonding with active-site residues, hydrophobic interactions, and modulation of residues associated with the mobile catalytic flap [9,10,39,40]. Catechol, pyrogallol, and galloyl functionalities are particularly relevant because neighboring oxygen donor groups can interact with the catalytic nickel ions [9,10]. Therefore, simultaneous presence of several structurally distinct phenolics in ME and MEW may permit additional interactions within or around the urease catalytic pocket. Nevertheless, the experimentally observed extract-level synergy cannot currently be assigned to individual metabolites, and testing of isolated compounds and defined combinations is required.

The antibacterial screening suggested greater preliminary biological activity in the polar fractions, particularly against *Bacillus cereus* and *Staphylococcus aureus* Gram-positive strains. However, several methodological factors must be considered. Differences in diffusion behavior in agar that may contribute to the apparent activity of different polarity extracts; larger inhibition zones cannot be attributed exclusively to intrinsic potency without quantitative broth-based MIC measurements; and translucent zones should not be interpreted as definitive evidence of bactericidal activity. Additionally, non-phenolic constituents or residual extraction solvents may contribute to these observed antibacterial effects. Regarding action mechanism, phenolic compounds and tannins have been associated with antibacterial effects through multiple pathways, including perturbation of membrane function, interference with energy metabolism, and modulation of bacterial signaling [41,42,43,44]. Future studies using broth microdilution, time-kill analysis, and mechanistic assays will be required in order to distinguish bacteriostatic from bactericidal effects as well as to clarify the specific contribution of individual metabolites. While our current study does not evaluate combination effects, the potential for synergy between polyphenols and conventional antimicrobial agents [45] represents a promising direction for future research regarding *L. guyonianum* constituents in combination-based antimicrobial strategies.

The molecular docking analysis provides structural support for the experimental urease-inhibition data while demonstrating the importance of multiple scoring parameters consideration. The composite CNN_VS metric was used as a ranking descriptor to identify ligands that satisfy both geometric pose reliability (CNNscore) and predicted binding strength (CNNaffinity), providing a robust computational tool for prioritizing candidate phytochemicals. Epigallocatechin achieved the highest CNN_VS among the phytochemicals, although AHA remained the highest-ranked ligand. In contrast, ranking according to Vina-derived binding energy favored larger compounds, particularly hesperidin, epigallocatechin gallate, epicatechin gallate, miquelianin, and quercetin. This divergence illustrates the different information encoded by conventional energy-based scoring, CNN-derived pose and affinity metrics [30]. The observed higher docking scores for larger glycosylated molecules may reflect a known size-related bias inherent in empirical scoring functions, such as those implemented in AutoDock Vina (v. 1.2.5), where an increased number of heavy atoms and potential hydrogen-bonding sites can lead to more additive interaction terms [30]. While this suggests potentially stronger predicted binding, it is important to consider that these scores may be influenced by the increased molecular complexity of the glycosides compared to their aglycone counterparts. Hesperidin represented the clearest example of this behavior, showing the most favorable mean Vina-derived binding energy but ranking only 14th according to CNN_VS.

The three-receptor approach revealed substantial conservation alongside target-dependent variability. Vina-derived binding energies were strongly correlated among the *H. pylori*, jack bean, and *S. pasteurii* ureases (ρ > 0.9), whereas CNN_VS rankings were considerably less transferable across receptor structures. This observation is significant because much of the previous urease docking literature has relied on a single receptor structure and often a single highest-ranked pose [46,47,48,49,50,51,52]. Also, previous studies have reported favorable docking of luteolin, kaempferol, quercetin, gallic acid, and structurally related phenolics against urease targets [46,49,50,53,54], and the present results provide further specificity. We consistently identified the four highest-ranked CNN_VS phytochemicals—epigallocatechin, epicatechin gallate, gallic acid, and epigallocatechin gallate—as top candidates across all three receptor models. Furthermore, these compounds exhibited highly consistent interaction modes, characterized by direct coordination with the catalytic Ni^+2^ ions in all representative poses (Table 10). However, while the identity of these key binders remained stable, their relative rankings varied depending on whether Vina-derived binding energy or CNN_VS scoring is used; this reflects the different priorities of thermodynamic affinity versus structural pose reliability. These findings suggest caution when assigning a unique “lead compound” based only on a single docking energy value.

Protein–ligand analysis contacts further highlighted the potential importance of the dinickel catalytic center. Hydrogen bonding represented the predominant interaction class across the three receptors, while direct metal coordination was also frequently observed. In particular, gallic acid, epigallocatechin, epicatechin gallate, and epigallocatechin gallate showed frequent interactions with nickel ions. The pyrogallol and galloyl motifs present in these compounds contain adjacent oxygen donor groups capable of adopting metal-binding geometries that are compatible with previously proposed mechanisms for phenolic urease inhibitors [55]. Especially, gallic acid showed reproducible metal engagement across the three receptor structures, despite a less favorable Vina-derived binding energy than several larger flavonoids. Conversely, hesperidin derived much of its predicted interaction profile from extensive hydrogen bonding rather than consistent direct engagement of the metal center. These contrasting patterns reinforce the need to differentiate predicted binding energy from mechanistically relevant active-site interactions.

A notable discrepancy exists between the experimental MEW extract IC_50_ values and individual docking rankings, as no single compound clearly exhibits the superior inhibitory potency that was observed in the 1:1 mixture. This suggests that the *L. guyonianum* anti-urease activity may result from the synergistic or additive effects of multiple phytochemicals acting within a complex matrix, rather than a single dominant metabolite action. Furthermore, while docking provides insights into individual binding potentials, it often fails to show multi-component dynamics or non-competitive/mixed inhibitory behaviors common among phenolics [49,56]. While many docking studies interpret active-site occupancy as competitive inhibition proof [46], such models may overlook more complex mechanisms involving metal coordination or interactions with residues that are involved in active-site-flap dynamics. This is reflected through divergence between our scoring metrics; while Vina-derived ΔG showed high consistency across targets (ρ = 0.910 for 1E9Y vs. 4H9M), CNN_VS rankings were significantly less consistent (ρ = 0.347, *p* = 0.134). This divergence suggests that while ΔG primarily captures general binding tendencies such as lipophilicity or molecular size, CNN_VS is more sensitive to the unique structural and geometric requirements of each specific receptor’s binding site. Consequently, these docking results should be viewed as structural hypotheses regarding how various constituents may interact with the catalytic dinickel center, providing a plausible mechanism of action that can guarantee further validation through purified-compound enzyme kinetics and experimental studies.

Additionaly, the *in silico* assessment indicates more that predicted urease-binding affinities may not always correlate with predicted oral drug-likeness profiles. Generally, lower-molecular-weight phenolic compounds and flavonoid aglycones displayed more favorable SwissADME profiles, including fewer Lipinski Rule-of-Five violations and greater predicted gastrointestinal absorption [57]. In contrast, glycosylated flavonoids and highly hydroxylated gallate derivatives showed increased TPSA and reduced predicted passive absorption. Thus, compounds such as hesperidin may exhibit favorable docking energies while displaying less favorable predicted oral permeability, whereas smaller aglycones may provide more balance between predicted physicochemical properties and predicted binding potential. Reduced systemic absorption should not necessarily be considered disadvantageous for compounds intended to act locally within the gastrointestinal tract. This is particularly relevant to our findings, as the most potent extract (MEW) was found to be rich in glycosylated flavonoids—compounds that SwissADME predicts—have low oral bioavailability and reduced permeability. This suggests that their anti-urease activity may be exerted through a local mechanism within the gastric lumen rather than via systemic circulation. However, it should be noted that gastrointestinal retention and local exposure cannot be inferred directly from SwissADME predictions alone [58,59].

Taken together, the experimental and computational findings point toward the polar fractions of *L. guyonianum* as the principal source of the observed anti-urease activity and identify several phenolic constituents that may contribute to this effect. Notably, the activity appears to emerge from a chemically complex mixture rather than from a single dominant metabolite, as supported by the synergistic behavior of ME and MEW as well as by the diversity of predicted urease-binding modes. However, the study nevertheless has several limitations as urease inhibition and antibacterial effects were evaluated at the extract level, preventing direct attribution of activity to individual compounds; antibacterial activity was assessed by disk diffusion rather than quantitative MIC determination; docking provides predicted rather than experimentally demonstrated binding modes; and the SwissADME analysis evaluates predicted ADME and drug-likeness properties rather than experimental pharmacokinetics or toxicity. Future investigations should therefore combine isolation or acquisition of the major candidate phytochemicals with purified-compound urease assays, kinetic characterization, defined combination studies, broth-based antibacterial testing, experimental assessment of pharmacokinetic and safety properties. In summary, this study provides an integrated perspective on the anti-urease potential of *L. guyonianum*, combining experimental bioassays with computational modeling to demonstrate that its polar fractions are a potent source of bioactive phytochemicals, that are able to interact effectively with the urease catalytic site.

## 4. Materials and Methods

### 4.1. Chemicals and Reagents

All chemicals, solvents, and analytical standards were purchased from Sigma-Aldrich (St. Louis, MO, USA), including petroleum ether, dichloromethane (CH_2_Cl_2_), methanol (CH_3_OH), Folin–Ciocalteu reagent (phosphomolybdic/phosphotungstic acid mixture), anhydrous sodium carbonate (Na_2_CO_3_), potassium iodate (KIO_3_), gallic acid (C_7_H_6_O_5_), catechin (C_15_H_14_O_6_), hydrochloric acid (HCl), urea (CH_4_N_2_O), phenol (C_6_H_6_O), sodium nitroprusside (Na_2_[Fe(CN)_5_NO]), phosphate buffer, sodium hydroxide (NaOH), sodium hypochlorite (NaOCl), dimethyl sulfoxide (DMSO, C_2_H_6_OS), Mueller–Hinton agar, and urease. Distilled water was used throughout the experimental procedures.

### 4.2. Plant Material and Extraction

Aerial parts of *Limoniastrum guyonianum* were collected during the flowering period in May 2023 from the southwestern region of Khenchela, eastern Algeria (34°49′3″ N, 6°46′23″ E), an area characterized by arid and saline environmental conditions. Botanical authentication was performed by Dr. Hallis Youcef at the Scientific and Technical Research Center for Arid Regions (CRSTRA). A voucher specimen was deposited in the CRSTRA herbarium under the identification code LG-AP-05/07. The collected plant material was shade-dried at room temperature for 15 days, mechanically ground, and sieved.

Sequential extraction was performed using solvents of increasing polarity, namely petroleum ether, dichloromethane, methanol, and a methanol–water mixture (5:1, *v*/*v*), with slight modifications to the previously reported procedure [60]. For extraction-yield determination, independent 50-g portions of dried plant powder were sequentially macerated in the respective solvents for 24 h with agitation at room temperature. The aqueous extraction was performed independently using 20 g of dried plant material in 0.5 L of distilled water for 72 h under periodic agitation [61]. The resulting organic filtrates were concentrated under reduced pressure using a rotary evaporator (Büchi, Flawil, Switzerland), whereas the aqueous extract was lyophilized to obtain a dry powder. The dried extracts were stored at 4 °C until further analysis while all assays were performed in technical triplicate.

### 4.3. Determination of Total Phenolic, Flavonoid, and Condensed Tannin Contents

Total phenolic content (TPC) was determined using the Folin–Ciocalteu method [62] with minor modifications. In a 96-well microplate, 20 µL of each extract solution (1 mg/mL) were mixed with 100 µL of Folin–Ciocalteu reagent diluted 1:10 and 75 µL of sodium carbonate solution (7.5%). The mixture was incubated in dark at room temperature for 2 h, after which absorbance was measured at 765 nm using a PerkinElmer EnSpire microplate reader (Singapore).

Gallic acid was used as reference standard over the concentration range of 25–200 µg/mL, yielding the calibration equation *y* = 0.0034*x* + 0.2205 (*R*^2^ = 0.962). Results were expressed as µg gallic acid equivalents per mg of extract (µg GAE/mg). All measurements were performed in triplicate. Total flavonoid content (TFC) was determined using the aluminum chloride colorimetric method [63]. Briefly, 100 µL of 2% AlCl_3_ solution were added to 100 µL of each extract solution (1 mg/mL), then an absorbance was measured at 430 nm after an incubation period of 10 min at room temperature.

Quercetin was used as the reference standard, yielding the calibration equation y = 0.0038x + 0.0272 (R^2^ = 0.9942). Results were expressed as µg quercetin equivalents per mg of extract (µg QE/mg) while all measurements were performed in technical triplicate.

Condensed tannin content (CTC) was determined using the vanillin–HCl method [64]. Note that, 25 µL of each extract solution (1 mg/mL in methanol) were mixed with 150 µL of vanillin solution (1% in methanol) and 75 µL of concentrated HCl (30%). After an incubation of 15 min, absorbance was measured at 500 nm. Catechin was used as the reference standard, yielding the calibration equation y = 0.026x + 0.0303 (R^2^ = 0.984), while the results were expressed as µg catechin equivalents per mg of extract (µg CE/mg) and all measurements were performed in technical triplicates.

### 4.4. LC-MS/MS Metabolite Profiling

Quantitative profiling was performed using the targeted LC–MS/MS method previously developed, optimized, and validated by Yilmaz et al. [65] for the simultaneous determination of 53 phytochemicals in medicinal and aromatic plants. The preparation and quantitative analysis of the extract samples in the present study followed the established protocol [65], conducted by the authors in accordance with the original method validation. No substantive modifications were made to the sample-preparation, chromatographic-separation, mass-spectrometric detection, calibration, or internal-standard procedures of the validated method. Before instrumental analysis, the extract samples were diluted to a final concentration of 1 mg/mL in methanol and filtered through 0.22 µm PTFE syringe filters.

In the original validation, the analyte-specific calibration ranges collectively spanned 0.02–50 mg/L. The reported coefficients of determination (r^2^) ranged from 0.957 to 0.999, with 51 of the 53 analytes showing r^2^ values of at least 0.991. Inter-day and intra-day precision, expressed as relative standard deviation, ranged from 0.69% to 2.51% and from 0.51% to 2.13%, respectively. The LODs and LOQs ranged from 1.3 to 237.5 µg/L and from 2.0 to 265.9 µg/L, respectively. Complete analyte-specific method-performance characteristics, including calibration equations and ranges, linearity, precision, recovery, LOD, LOQ, and expanded measurement uncertainty, are provided in Supplementary Table S1 and in the original validation study [65].

The validated method employed three deuterated surrogate internal standards—ferulic acid-d_3_, rutin-d_3_, and quercetin-d_3_—which were assigned to defined analyte groups according to the grouping scheme established in the original method [65]. Because matched isotopically labelled standards were not available for every analyte, these three compounds should be regarded as group-specific surrogate standards rather than exact isotope-labelled analogues for the entire analyte panel. Consequently, complete correction of analyte- and matrix-specific ion suppression or enhancement cannot be assumed. No additional analyte-specific matrix-effect assessment was performed for the extracts examined in the present study, and this limitation should be considered when interpreting the quantitative results.

### 4.5. In Vitro Urease Inhibition Assay

Urease inhibitory activity was assessed using the indophenol blue method [38,66]. Extracts were tested at concentrations ranging from 3.125 to 200 µg/mL. A jack bean urease preparation (1 mg/mL in 0.12 M phosphate buffer, pH 8.2) with a reported nominal activity concentration of 5 U/mL was used. In a 96-well microplate, 10 µL of each extract solution was mixed with 50 µL of urea solution (100 mM) and 25 µL of the urease preparation. Thus, each well received a nominal urease activity of 0.125 U, corresponding to approximately 1.47 U/mL in the initial 85 µL reaction mixture. The enzyme activity was not independently determined under the present assay conditions. The same urease preparation and nominal activity concentration were used throughout all experiments.

After incubation at 30 °C for 15 min, 45 µL of phenol reagent (1% phenol containing 0.005% sodium nitroprusside, *w*/*v*) and 70 µL of alkaline reagent containing NaOH and sodium hypochlorite (approximately 0.97 g/L available chlorine) were added sequentially. The plates were then incubated at 30 °C for 50 min, and absorbance was measured at 630 nm. Samples were analyzed in technical triplicate, with one extract-specific blank included for each sample concentration. The absorbance of the corresponding blank was subtracted separately from each of the three sample absorbance values to correct for intrinsic extract coloration.

Urease inhibition was calculated according to Equation (1):

(1) $$Inhibition\left(\%\right)=\left(1-\frac{A_{sample}}{A_{control}}\right)\times 100$$

where $A_{sample}$ and $A_{control}$ represent the absorbance values of the sample and control reactions, respectively. Thiourea was used as the positive control, and all assays were performed in technical triplicate.

Dose–response curves were generated by plotting percentage urease inhibition against log-transformed extract concentrations. IC_50_ values were estimated by nonlinear regression using a five-parameter logistic (5PL) model. Fit goodness was evaluated using determination coefficient (*R*^2^), residual analysis, and confidence intervals associated with the estimated IC_50_ values.

Interactions between ME and MEW extracts were evaluated according to Chou–Talalay approach [67]. Dose–response curves were generated individually for each extract and for their fixed 1:1 combination. Values of combination index (CI) were calculated over fractional effect levels (*Fa*) ranging from 0.10 to 0.80 according to Equation (2):

(2) $$CI\left(Fa\right)=\frac{f_{ME}D_{mix}\left(Fa\right)}{D_{ME}\left(Fa\right)}+\frac{f_{MEW}D_{mix}\left(Fa\right)}{D_{MEW}\left(Fa\right)}$$

where $f_{ME}\wedge f_{MEW}$ represent the fractional contributions of the ME and MEW extracts, respectively; $D_{mix}\left(Fa\right)$ is the total dose of the mixture required to produce a given fractional effect; $D_{ME}\left(Fa\right)$ and $D_{MEW}\left(Fa\right)$ are the doses of the individual extracts required to produce the same effect. For a fixed 1:1 mixture: $f_{ME}=f_{MEW}=0.5$. CI values < 1, =1, and >1 were interpreted as synergistic, additive, and antagonistic interactions, respectively [67].

The dose-reduction index (DRI) for each component was calculated according to Equation (3):

(3) $$DRI_{i}\left(Fa\right)=\frac{D_{i}\left(Fa\right)}{f_{i}D_{mix}\left(Fa\right)}$$

where $D_{i}\left(Fa\right)$ is the dose of component (i) required to achieve a given fractional effect when used alone, whereas $f_{i}D_{mix}\left(Fa\right)$ represents the corresponding dose of that component within the combination. A DRI value > 1 indicates a reduction in the dose required when the component is used in combination.

Combination-index and dose-reduction analyses were performed according to the Chou–Talalay median-effect principle using CompuSyn v1.0. CI confidence intervals were estimated by non-parametric cluster bootstrap using the percentile method applied to the triplicate datasets; resampling and percentile calculations were performed in NumPy, with SciPy refitting for each bootstrap resample. Effect levels requiring extrapolation beyond the experimentally tested concentration range were not used for the primary interpretation of combination effects.

### 4.6. Antibacterial Assay 

The antibacterial activity of the five extracts (PE, DCM, ME, MEW 5:1 *v*/*v*, and AQ) was evaluated using an agar disk diffusion assay adapted for crude botanical extracts [68,69]. Four reference bacterial strains were tested: the Gram-positive strains *Bacillus cereus* and *Staphylococcus aureus*, and the Gram-negative strains *Escherichia coli* and *Salmonella enteritidis*.

Mueller–Hinton agar was poured into sterile Petri dishes (approximately 20 mL per plate). Bacterial suspensions prepared from 18–24 h cultures were adjusted to a 0.5 McFarland standard (approximately 1 × 10^8^ CFU/mL) in sterile saline and uniformly inoculated onto the agar surface.

Extract solutions were prepared in DMSO 99% purity for the apolar fractions or DMSO–water (1:4, *v*/*v*) for the polar fractions, and filtered through 0.22 µm PTFE syringe filters. The tested concentrations were 25, 12.5, 6.25, and 3.125 mg/mL. Whatman filter-paper disks (5 mm diameter), used for the plant extracts, were impregnated with 10 µL of each solution, corresponding to extract loads of 250, 125, 62.5, and 31.25 µg/disk, respectively. The final concentration of DMSO in the test solutions corresponded to approximately 9.9 µL of DMSO for the apolar extracts and 1.98 µL of DMSO for the polar extracts.

The disks were air-dried for 30 min under a sterile laminar-flow hood and put onto the inoculated agar plates. Commercial gentamicin (CN10, 10 µg/disk) and cefotaxime (CT10, 10 µg/disk) disks (6 mm diameter) were used as positive controls, whereas to account for potential solvent-induced inhibition, one DMSO vehicle control was used. Using DMSO, and 10 µL was applied per 5-mm disk with a concentration of 9.9 µL of DMSO.

Plates were allowed to stand for 15 min to allow diffusion and were subsequently incubated at 37 °C for 24 h. Inhibition zones were measured as the total diameter, including the disk, using a calibrated digital caliper [68]. Accordingly, a value equal to the 5-mm diameter of an extract disk indicated no measurable extension of the inhibition zone beyond the disk edge. Clear or translucent regions observed within the inhibition halo were recorded separately as translucent zones. These regions were not interpreted as evidence of membrane disruption or bacteriolysis because no direct mechanistic assay of bacterial lysis was performed.

Experiments were performed in technical triplicates, and results are reported as mean ± SD.

### 4.7. Molecular Docking

#### 4.7.1. Protein Preparation

Three crystallographic urease structures were retrieved from the Protein Data Bank (PDB): *Helicobacter pylori* urease (PDB ID: 1E9Y; resolution, 3.00 Å) [70,71], jack bean (*Canavalia ensiformis*) urease (PDB ID: 4H9M; resolution, 1.52 Å) [72], and *Sporosarcina pasteurii* urease (PDB ID: 4UBP; resolution, 1.55 Å) [73,74]. While the experimental inhibition assays were conducted using Jack bean urease, multiple crystallographically characterized ureases from different organisms were selected for docking to provide a broader computational perspective on potential binding modes across diverse urease structures. All three structures contain the conserved dinickel catalytic center and were available in complex with the reference urease inhibitor acetohydroxamic acid (AHA).

Receptor structures were downloaded directly from the RCSB Protein Data Bank. During preprocessing, ANISOU records, crystallographic water molecules (HOH/WAT/DOD), and the co-crystallized AHA ligand were removed. Catalytically relevant carbamylated lysine residues (KCX219 in 1E9Y, KCX490 in 4H9M, and KCX220 in 4UBP) and the catalytic Ni^2+^ ions were retained. The presence of at least two Ni^2+^ ions and the corresponding KCX residue was checked for each receptor, together with a Ni–Ni distance within 2.5–4.2 Å. Hydrogen atoms were added and protonation states were assigned at pH 7.4 using Open Babel. The crystallographic AHA coordinates were retained separately for subsequent redocking and RMSD validation [75]. The catalytically relevant carbamylated lysine residues (KCX219 in 1E9Y, KCX490 in 4H9M, and KCX220 in 4UBP) were retained in their modified states [76]. The co-crystallized AHA ligand was removed from each receptor and retained for subsequent redocking validation.

#### 4.7.2. Ligand Library

Molecular structures and SMILES strings for 20 selected phytochemicals quantified in *L. guyonianum*, together with the reference urease inhibitor AHA, were retrieved from PubChem [77] (accessed on 14 June 2026). Ligand structures were retrieved from PubChem through PUG-REST as three-dimensional SDF files when available, with two-dimensional/SMILES-based retrieval used as a fallback. Three-dimensional structures were generated or completed using Open Babel with Gasteiger partial charges, followed by geometry optimization using GFN2-xTB under tight convergence criteria. CM5 atomic charges were subsequently obtained from the optimized structures. The PubChem AHA structure was prepared while preserving its three-dimensional geometry for redocking validation. To obtain a computationally tractable and broadly comparable small-molecule ligand set, an extended molecular-weight range was adopted to retain naturally occurring flavonoid glycosides such as hesperidin, despite its molecular weight exceeding the conventional 500 Da threshold. Tannic acid, although previously reported to display anti-urease activity [78], was excluded from the docking library because of its substantially higher molecular weight (~1701 Da) and extensive conformational flexibility, which make it poorly suited to the small-molecule docking workflow adopted in this study [79].

The selection of compounds for detailed interaction analysis was based on a dual-criteria approach: we prioritized phytochemicals that were among the most abundant constituents quantified via LC-MS/MS and which also demonstrated superior docking performance (high CNN_VS scores). This ensured that our structural interpretations will focuse on the most biologically relevant candidates likely to contribute to the observed activity.

#### 4.7.3. Docking Protocol

Molecular docking calculations were performed using GNINA version 1.3 [80] on an NVIDIA GeForce RTX 3090 GPU (24 GiB). CNN-based scoring was enabled, with exhaustiveness set to 64, 30 output modes generated per docking run, and CNN rotation sampling set to 15 iterations [30]. The docking search space was defined using GNINA’s --autobox_ligand option around the crystallographic AHA binding site with the default 4 Å padding. It should be noted that this approach focuses the search on the known catalytic region and may not account for alternative or allosteric binding sites. The resulting grid centers (x, y, z; Å) were 127.863, 126.349, 87.546 for 1E9Y; 18.782, −57.810, −24.152 for 4H9M; and 29.274, 73.051, 72.786 for 4UBP. For each phytochemical–receptor pair, 20 independent docking runs were performed using different random seeds to evaluate pose reproducibility and scoring stability. The top-ranked pose from each independent run was retained for subsequent analysis. Considering the three urease targets; therefore each phytochemical was evaluated across 60 retained poses (20 runs × 3 receptors). AHA was subjected to the same 20 independent redocking runs per receptor, generating 60 validation poses in total. The highest-ranked validation pose for each receptor was used for RMSD and CNNscore assessment against the crystallographic reference pose. GNINA provides three complementary scoring parameters: the Vina-derived docking score (kcal·mol^−1^), CNNscore, and CNNaffinity. CNNscore is a dimensionless metric ranging from 0 to 1 that reflects the predicted quality of the generated binding pose, whereas CNNaffinity provides a neural-network-based estimate of ligand binding affinity on a pK-like scale [30]. For comparative purposes, Vina-derived energy score (ΔG) was converted into an apparent dissociation constant and the corresponding calculated pK value according to Equation (4):

(4) $$K_{calc}=exp\left(\frac{\Delta G}{RT}\right),{pK}_{calc}=-log^{10}\left(K_{calc}\right)=\frac{-\Delta G}{RTln10}$$

with R = 0.0019872036 kcal·mol^−1^·K^−1^ and T = 298 K.

Compounds were ranked using the virtual-screening metric CNN_VS [30], calculated for each pose as:

(5) CNNVS=CNNscore×CNNaffinity

To assess the consistency of ligand interaction potential across diverse targets, CNN_VS values were averaged across all 60 retained poses for each phytochemical (comprising 20 independent runs per receptor across 3 distinct urease structures). This averaging approach was set to emphasize reproducible performance and cross-target robustness rather than absolute thermodynamic affinity or individual high-scoring poses. A threshold of pK_calc ≥ 6.30, calculated from the Vina-derived ΔG, was used for active/inactive classification as per the adopted computational framework [30]. Redocking of the crystallographic AHA ligand was used to assess docking-protocol reliability. Validation was considered successful when the RMSD between the predicted and crystallographic AHA poses was ≤2.0 Å. Receptor suitability was additionally assessed using a CNNscore threshold of ≥0.90 for the redocked co-crystallized ligand [30].

### 4.8. Interaction Analysis

Non-covalent protein–ligand interactions were analyzed using the Protein–Ligand Interaction Profiler (PLIP, version 2.3.0) [81]. No binding site was manually predefined in PLIP; interactions were identified using the program’s standard geometric criteria. Particular attention was given to interactions within the urease catalytic site. Metal-coordination distances between ligand donor atoms (O or N) and the catalytic Ni^2+^ ions were evaluated using a cutoff distance of 3.5 Å. Ni^2+^-coordinating poses percentage was calculated as: (number of poses with at least one ligand donor atom ≤ 3.5 Å from either Ni ion)/total evaluated poses × 100. Six interaction classes were considered: hydrogen bonds, metal coordination, hydrophobic contacts, salt bridges, π-stacking interactions, and π-cation interactions. Representative two-dimensional protein–ligand interaction diagrams were generated using BIOVIA Discovery Studio Visualizer version 2025.

### 4.9. Statistical Analysis and Synergy Modeling

Dose–response curves were generated via plotting percentage urease inhibition against extract concentration on a logarithmic scale. Individual replicate measurements were fitted using bounded nonlinear least-squares regression with a five-parameter logistic (5PL) model:

(6) $$E(x)=d+\frac{a-d}{{\left[1+{\left(\frac{x}{c}\right)}^{b}\right]}^{g}}$$

where E(x) is the percentage inhibition at concentration x; while a and d  are the lower and upper response asymptotes, respectively; b is the Hill slope; c is the inflection concentration; and g is the asymmetry parameter. Multiple initial parameter values were evaluated, and the converged solution with the lowest residual sum of squares was retained. The IC_50_ was defined as the concentration predicted to produce 50% inhibition of uninhibited urease activity, obtained via analytical inversion of the fitted 5PL curve.

Fit goodness was evaluated using the coefficient of determination (*R*^2^), adjusted *R*^2^, root-mean-square error, residual analysis, and bootstrap confidence intervals for the IC_50_ evaluations.

The interaction between ME and MEW was evaluated exclusively at the fixed tested 1:1 concentration ratio. At each affected fraction (Fa), the concentrations of ME, MEW, and the mixture predicted to produce the same inhibition level were obtained via analytical inversion of their respective 5PL curves. The combination index (CI) was calculated relative to Loewe additivity as:

(7) $$CI\left(Fa\right)=\frac{0.5\;D_{mix}\left(Fa\right)}{D_{ME}\left(Fa\right)}+\frac{0.5\;D_{mix}\left(Fa\right)}{D_{MEW}\left(Fa\right)}$$

where $D_{mix}$ is the total concentration of the 1:1 mixture, while $D_{ME}$ and $D_{MEW}$ are the concentrations of the individual extracts predicted to produce the same effect. CI < 1, CI = 1, and CI > 1 values indicate deviation toward synergism, additivity, and antagonism, respectively. Accordingly, interactions’ conclusions were restricted to the tested 1:1 ratio and were not generalized to other mixture proportions. The Fa range was restricted to 0.10–0.40 to ensure that calculations were performed within the linear portion of the dose-response curve.

Uncertainty in the IC_50_ and CI evaluations was assessed using a stratified nonparametric bootstrap. Replicate observations were sampled with replacement within each concentration level while retaining the original number of observations per concentration. The 5PL curves were refitted and the corresponding IC_50_ and CI values recalculated for 2000 bootstrap iterations. The 95% confidence intervals were defined by the 2.5th and 97.5th percentiles of the valid bootstrap estimates.

CI values calculated at prespecified effect levels were considered as model-derived because all corresponding doses were obtained from the fitted 5PL curves. For CI values calculated at experimentally tested mixture concentrations, the mixture concentration and mean inhibition were experimentally observed, whereas the equieffective concentrations of the individual extracts remained model-derived. Extrapolated values—when the target effect or any required inverse dose fell outside the corresponding experimentally tested concentration or observed-response range—were not used as primary evidence of synergy.

Because only 3 replicates were available per concentration, the precision of the fitted curves and bootstrap intervals was limited. Therefore, CI results were interpreted cautiously. Synergistic interactions were considered significant if the entire 95% CI remained below 1 within the experimentally supported range; if the interval included 1, the interaction was deemed statistically inconclusive relative to additivity.

Experimental data are presented as mean ± SD unless otherwise specified. Prior to performing one-way analysis of variance (ANOVA), the normality assumptions and variance homogeneity were assessed using Shapiro-Wilk and Levene’s tests, respectively. Only datasets meeting these criteria were subjected to Tukey’s honestly significant difference (HSD) post hoc testing. Statistical significance was established at *p* < 0.05.

For the molecular docking dataset, monotonic associations among scoring parameters were evaluated using Spearman’s rank correlation coefficient. Statistical analyses were performed in Python 3.11 using SciPy v1.11 and Statsmodels v0.14.

### 4.10. In Silico ADME and Drug-Likeness Assessment

The predicted pharmacokinetic properties and drug-likeness of the quantified phytochemicals were evaluated using SwissADME [82]. The analyzed parameters included Lipinski’s Rule-of-Five violations, topological polar surface area (TPSA), lipophilicity expressed as Consensus Log P, gastrointestinal absorption, blood–brain barrier permeability, P-glycoprotein substrate status, predicted inhibition of major cytochrome P450 isoforms (CYP1A2, CYP2C9, CYP2C19, CYP2D6, and CYP3A4), and bioavailability score. Consensus Log P was derived from the lipophilicity prediction methods implemented in SwissADME. The brain or intestinal estimated permeation predictive model (BOILED-Egg) was additionally used to visualize predicted gastrointestinal absorption and blood–brain barrier permeation in accordance with [83].

## 5. Conclusions

This study provides the first integrated evaluation of the anti-urease potential of *Limoniastrum guyonianum* through combined phytochemical, biological, and computational approaches. Targeted LC–MS/MS profiling showed that ME and MEW extracts were the richest in quantified phytochemicals, particularly phenolic acids and flavonoids, revealing that these fractions also displayed the strongest urease-inhibitory activity.

The 1:1 combination of ME and MEW extracts showed synergistic urease inhibition, suggesting that the observed activity is likely associated with the combined contribution of multiple constituents rather than a single dominant compound. The same polar fractions also exhibited the most relevant preliminary antibacterial effects, particularly against Gram-positive strains.

Molecular docking provided a structural hypothesis for this activity, suggesting the potential involvement of several phenolic constituents in urease binding, with epigallocatechin, epicatechin gallate, gallic acid, and epigallocatechin gallate among the highest-ranked phytochemicals according to CNN_VS metric. Their frequent interactions with the catalytic dinickel center suggest a plausible mechanism for the observed activity; however, as the docking search was localized exclusively to that site, alternative binding modes cannot be excluded, and these computational predictions require further validation through enzyme kinetic studies using isolated compounds.

SwissADME analysis further highlighted substantial differences in predicted drug-likeness and gastrointestinal absorption among the quantified phytochemicals, with lower-molecular-weight aglycones that generally exhibit more favorable predicted oral pharmacokinetic properties than glycosylated and highly hydroxylated compounds.

Overall, *L. guyonianum* represents a promising source of phenolic constituents with urease-inhibitory and antibacterial potential. Further studies should focus on purified compounds, defined compound combinations, quantitative antibacterial assays, enzyme kinetics, experimental pharmacokinetics, and safety evaluation to validate the biological relevance of the present findings.

## Figures and Tables

**Table 1:** Extraction yields of *Limoniastrum guyonianum* aerial-part extracts obtained using organic solvents of increasing polarity alongside independently prepared aqueous extract.

Extract	Yield (%)	95% CI
Petroleum ether (PE)	1.21 ± 0.05 ^d^	1.09–1.33
Dichloromethane (DCM)	0.67 ± 0.06 ^e^	0.51–0.82
Methanol (ME)	3.74 ± 0.11 ^c^	3.48–4.00
Methanol–water (MEW)	4.16 ± 0.13 ^b^	3.84–4.48
Aqueous (AQ)	16.35 ± 0.22 ^a^	15.81–16.89

Values are expressed as mean ± SD of three independent extractions (*n* = 3). Extraction yield (%) was calculated relative to the initial dry plant mass. Different superscript letters indicate significant differences among extracts according to one-way ANOVA followed by Tukey’s HSD test (*p* < 0.05). 95% CI, 95% confidence interval. PE, petroleum ether; DCM, dichloromethane; ME, methanol; MEW, methanol–water (5:1, *v/v*); AQ, aqueous extract.

**Table 2:** Total phenolic, flavonoid, and condensed tannin contents of *Limoniastrum guyonianum* extracts using organic solvents of increasing polarity.

Extract	TPC (µg GAE/mg)	TFC (µg QE/mg)	CTC (µg CE/mg)
Petroleum ether (PE)	43.51 ± 4.86 ^d^	23.67 ± 0.70 ^d^	10.57 ± 0.32 ^c^
Dichloromethane (DCM)	57.46 ± 4.34 ^c^	85.67 ± 2.17 ^c^	16.67 ± 0.85 ^b^
Methanol (ME)	157.65 ± 4.40 ^b^	223.62 ± 10.64 ^a^	29.83 ± 2.11 ^a^
Methanol–water (MEW)	222.39 ± 0.61 ^a^	114.34 ± 16.82 ^b^	27.80 ± 1.22 ^a^

Values are expressed as mean ± SD of three independent measurements (*n* = 3). TPC, total phenolic content, expressed as µg gallic acid equivalents per mg of extract (µg GAE/mg); TFC, total flavonoid content, expressed as µg quercetin equivalents per mg of extract (µg QE/mg); CTC, condensed tannin content, expressed as µg catechin equivalents per mg of extract (µg CE/mg). Different superscript letters within the same column indicate significant differences among extracts according to one-way ANOVA followed by Tukey’s HSD test (*p* < 0.05).

**Table 3:** Targeted LC–MS/MS quantification of phytochemicals detected in *Limoniastrum guyonianum* extracts, with selected chromatographic, MRM, and sensitivity parameters.

SI ID	Analyte	RT (min)	MRM Precursor Ion (*m*/*z*)	Product Ion(s) (*m*/*z*)	LOD/LOQ (µg/L)	PE (µg/g)	DCM (µg/g)	ME (µg/g)	MEW (µg/g)
1	Quinic acid ^†^	3.0	190.8	93.0	25.7/33.3	N.D.	N.D.	463 ± 17	1322 ± 49
4	Gallic acid	4.4	168.8	79.0	13.2/17.0	N.D.	N.D.	3396 ± 38	4518 ± 51
5	Epigallocatechin	6.7	304.8	219.0	237.5/265.9	N.D.	N.D.	N.D.	4267 ± 79
6	Protocatechuic acid	6.8	152.8	108.0	21.9/38.6	N.D.	37 ± 1	717 ± 25	389 ± 14
10	Protocatechuic aldehyde ^†^	8.5	137.2	92.0	15.4/22.2	N.D.	N.D.	308 ± 12	132 ± 5
11	Tannic acid ^†^	9.2	182.8 ^a^	78.0	15.3/22.7	N.D.	N.D.	233 ± 4	327 ± 6
12	Epigallocatechin gallate	9.4	457.0	305.1	61.0/86.0	N.D.	N.D.	391 ± 6	1333 ± 20
17	Caffeic acid	12.1	179.0	134.0	7.7/9.5	N.D.	22.0 ± 0.3	3525 ± 54	852 ± 13
20	Syringic aldehyde ^†^	14.6	181.0	151.1	19.7/28.0	N.D.	N.D.	N.D.	35.0 ± 0.8
22	Epicatechin gallate ^†^	15.5	441.0	289.0	19.5/28.5	53 ± 1	N.D.	N.D.	N.D.
24	*p*-Coumaric acid	17.8	163.0	93.0	25.9/34.9	N.D.	N.D.	555 ± 11	146 ± 3
31	Miquelianin	24.1	477.0	150.9	10.6/14.7	N.D.	36.0 ± 0.8	4054 ± 89	5333 ± 117
34	Isoquercitrin ^†^	25.6	463.0	271.0	8.7/13.5	N.D.	N.D.	1733 ± 38	1000 ± 22
35	Hesperidin ^†^	25.8	611.2	449.0	19.0/26.0	N.D.	N.D.	233 ± 8	178 ± 6
47	Quercetin	35.7	301.0	272.9	15.5/19.0	N.D.	N.D.	383 ± 7	216 ± 4
48	Naringenin ^†^	35.9	270.9	119.0	2.6/3.9	N.D.	6.0 ± 0.2	63 ± 2	23 ± 1
49	Hesperetin ^†^	36.7	301.0	136.0, 286.0	7.1/9.1	N.D.	N.D.	25 ± 1	13.0 ± 0.4
50	Luteolin ^†^	36.7	284.8	151.0, 175.0	2.6/4.1	N.D.	6.0 ± 0.2	N.D.	N.D.
52	Kaempferol	37.9	285.0	239.0	10.2/15.4	N.D.	N.D.	13.0 ± 0.3	N.D.
53	Apigenin	38.2	268.8	151.0, 149.0	1.3/2.0	N.D.	13.0 ± 0.2	N.D.	N.D.
56	Acacetin ^†^	40.7	283.0	239.0	1.5/2.5	3.0 ± 0.1	328 ± 12	34 ± 1	24.0 ± 0.9

Quantitative values are the means of three determinations (*n* = 3) and are reported as concentration ± expanded measurement uncertainty (*k* = 2, approximately 95% coverage) in µg/g dry extract. The expanded uncertainty associated with each concentration was calculated as $\;U_{calculated}=C\times U$, where C is the measured concentration and U is the relative expanded uncertainty. RT, retention time; MRM, multiple reaction monitoring; LOD, limit of detection; LOQ, limit of quantification; N.D., not detected (signal below the established LOD); PE, petroleum ether extract; DCM, dichloromethane extract; ME, methanolic extract; MEW, methanol–water extract (5:1, *v/v*). MRM acquisition was performed in negative ESI mode for all analytes listed except hesperidin, which was acquired in positive ESI mode. ^a^ For tannic acid, *m*/*z* 182.8 corresponds to a low-mass galloyl-derived diagnostic ion associated with the commercial tannic acid standard rather than the intact molecular precursor; quantification was nevertheless performed directly against the commercial tannic acid standard using the corresponding MRM response. ^†^ Compounds quantitatively reported for the first time in *L. guyonianum* in the present study, to the best of our knowledge. S1 ID refers to the numbering used in Supplementary Table S1, which contains the complete validation dataset for 53 target analytes and three deuterated internal standards.

**Table 4:** Urease inhibitory activity of *Limoniastrum guyonianum* extracts and combination analysis of the methanolic and hydromethanolic fractions.

Extract/Fraction	IC_50_ (µg/mL)	Inhibition % (at 200 µg/mL)	95% CI (µg/mL)	CI Range ^a^
Petroleum ether (PE)	NR (> 200)	—	—	—
Dichloromethane (DCM)	NR (> 200)	—	—	—
Methanol (ME)	NR (> 200)	44.29 ± 1.12	—	—
Methanol–water (MEW)	54.43 ± 4.70	82.26 ± 2.52	45.64–64.08	—
ME + MEW (1:1)	44.66 ± 1.21	84.80 ± 2.13	42.14–46.89	0.518–0.689
Aqueous (AQ)	NR (> 200)	—	—	—
Thiourea (positive control)	19.62 ± 0.34	96.72 ± 1.01	18.86–20.19	—

IC_50_ values are expressed as mean ± SD from three independent measurements (*n* = 3) and were estimated by five-parameter logistic (5PL) regression. NR, IC_50_ not reached within the experimentally tested concentration range (3.125–200 µg/mL). PE, petroleum ether extract; DCM, dichloromethane extract; ME, methanolic extract; MEW, methanol–water extract (5:1, *v/v*); AQ, aqueous extract; CI, combination index; R^2^, coefficient of determination of the fitted dose–response model. ^a^ CI combination index for the fixed 1:1 ME–MEW combination reported over the non-extrapolated fractional-effect range (Fa = 0.10–0.40); CI < 1 indicates synergism. Over this range, dose-reduction index (DRI) values were ≥8.24 for ME and ≥1.75 for MEW. The complete Fa–CI–DRI dataset, including bootstrap confidence intervals, is provided in Supplementary Table S2. Inhibition percentage at the highest experimentally tested concentration (200 µg/mL).

**Table 5:** Inhibition and translucent zone diameters of tested bacterial strains using various *L. guyonianum* extracts at different concentrations (mg/mL). Results are presented for: PE (petroleum ether), DCM (dichloromethane), ME (methanol), MEW (methanol–water, 5:1 *v*/*v*), AQ (aqueous extract) and solvent controls (DMSO). C. indicates the concentration used for testing. ^†^ Lysis zones for ME: 8.00 mm and MEW: 9.00 mm at the highest tested concentration (25 mg/mL). n.i refers to no inhibition zone.

Bacteria	C. (mg/mL)	Inhibition Zones (mm)							
		PE	DCM	ME	MEW	AQ	CN10	CT10	DMSO
*Bacillus cereus* ATCC 10876	25	6.30 ± 0.15	n.i	8.00 ± 0.00	18.50 ± 0.07	7.00 ± 0.00	15	0	0
	12.5	n.i	n.i	6.00 ± 0.00	13.50 ± 0.07	6.00 ± 0.00			
	6.25	n.i	n.i	6.00 ± 0.00	12.50 ± 0.07	n.i			
	3.125	n.i	n.i	n.i	12.50 ± 0.00	n.i			
*Escherichia coli* ATCC 25922	25	7.00 ± 0.00	n.i	n.i	8.00 ± 0.00	n.i	13	9	0
	12.5	6.00 ± 0.00	n.i	n.i	n.i	n.i			
	6.25	5.00 ± 0.00	n.i	n.i	n.i	n.i			
	3.125	5.00 ± 0.00	n.i	n.i	n.i	n.i			
*Staphylococcus aureus* ATCC 25923	25	8.00 ± 0.00	15.50 ± 0.07	18.50 ± 0.07 ^†^	19.50 ± 0.07 ^†^	8.00 ± 0.00	15	0	0
	12.5	6.00 ± 0.00	8.00 ± 0.00	14.00 ± 0.14	16.50 ± 0.07	7.00 ± 0.00			
	6.25	5.00 ± 0.00	6.00 ± 0.00	8.50 ± 0.07	15.50 ± 0.07	n.i			
	3.125	n.i	n.i	7.00 ± 0.00	9.50 ± 0.35	n.i			
*Salmonella enteritidis* ATC 13076	25	6.00 ± 0.00	n.i	n.i	9.00 ± 0.00	6.00 ± 0.00	16	11	0
	12.5	n.i	n.i	n.i	n.i	n.i			
	6.25	n.i	n.i	n.i	n.i	n.i			
	3.125	n.i	n.i	n.i	n.i	n.i			

**Table 6:** Summary docking statistics and target validation for the three urease receptors. Values are averaged across all retained poses for each receptor. The AHA CNN score is the receptor-validation metric considered by Buccheri et al. [30].

Parameter	1E9Y	4H9M	4UBP
AHA CNN score (validation)	0.933	0.906	0.940
AHA pose 1 RMSD (Å)	1.360	1.669	1.669
Mean CNN score	0.661	0.551	0.647
Mean CNN affinity	4.768	4.304	4.633
Median binding energy ∆G (kcal·mol^−1^)	−6.73	−7.13	−7.23
Mean contacts per pose	6.27	6.84	6.91
Poses coordinating Ni (%)	86.4	79.6	63.9

**Table 7:** Cross-receptor docking and interaction metrics for the 20 phytochemicals quantified in *Limoniastrum guyonianum*.

CNN_VS Rank	Compound	Analyte ID	CNN Score	CNN Affinity	CNN_VS	ΔG kcal·mol^−1^	ΔG Rank	pK Calc	ΔG_best (kcal·mol^−1^)	Ni-Coordinating Poses (%)	Contacts Pose
1	Epigallocatechin	5	0.749 ± 0.05	5.08 ± 0.21	3.80 ± 0.31	−7.55 ± 0.33	9	5.53	−8.04	83.3	8.48
2	Epicatechin gallate	22	0.636 ± 0.17	5.61 ± 0.29	3.61 ± 1.09	−8.89 ± 0.41	3	6.52	−9.76	86.7	8.45
3	Gallic acid	4	0.834 ± 0.05	4.21 ± 0.08	3.52 ± 0.22	−6.36 ± 0.29	14	4.67	−6.73	100.0	6.68
4	Epigallocatechin gallate	12	0.615 ± 0.18	5.50 ± 0.31	3.44 ± 1.14	−9.08 ± 0.45	2	6.66	−9.83	86.7	9.17
5	Acacetin	56	0.644 ± 0.06	4.74 ± 0.31	3.07 ± 0.47	−6.99 ± 0.52	11	5.13	−8.32	38.3	6.68
6	Quercetin	47	0.630 ± 0.04	4.81 ± 0.31	3.04 ± 0.35	−8.82 ± 0.34	5	6.46	−9.16	100.0	6.72
7	Protocatechuic acid	6	0.803 ± 0.05	3.74 ± 0.14	3.00 ± 0.14	−6.13 ± 0.18	15	4.49	−6.30	100.0	8.08
8	Naringenin	48	0.606 ± 0.08	4.93 ± 0.20	3.00± 0.51	−6.97 ± 0.37	12	5.11	−7.56	31.7	6.38
9	Apigenin	53	0.621 ± 0.07	4.67 ± 0.30	2.91 ± 0.46	−6.89 ± 0.49	13	5.05	−7.58	41.7	6.33
10	Protocatechuic aldehyde	10	0.835 ± 0.02	3.46 ± 0.08	2.89 ± 0.06	−5.45 ± 0.18	17	3.99	−5.72	100.0	4.55
11	Isoquercitrin	34	0.527 ± 0.18	5.04 ± 0.33	2.70 ± 1.02	−8.69 ± 0.73	6	6.37	−9.56	66.7	7.52
12	Luteolin	50	0.582 ± 0.04	4.57 ± 0.23	2.67 ± 0.29	−8.24 ± 0.41	7	6.04	−8.74	66.7	6.83
13	Hesperetin	49	0.526 ± 0.08	4.94 ± 0.29	2.61 ± 0.52	−7.05 ± 0.38	10	5.17	−7.83	53.3	5.77
14	Hesperidin	35	0.414 ± 0.08	5.81 ± 0.51	2.44 ± 0.67	−9.62 ± 0.45	1	7.05	−10.38	70.0	9.57
15	Syringic aldehyde	20	0.583 ± 0.09	4.11 ± 0.42	2.41 ± 0.51	−4.86 ± 0.38	19	3.56	−5.52	70.0	4.12
16	Miquelianin	31	0.458 ± 0.17	5.14 ± 0.43	2.41 ± 1.06	−8.83± 0.90	4	6.47	−9.86	75.0	8.68
17	Caffeic acid	17	0.634 ± 0.09	3.65 ± 0.55	2.29 ± 0.32	−4.60 ± 0.54	20	3.37	−5.56	6.7	3.45
18	Quinic acid	1	0.552 ± 0.05	4.06 ± 0.36	2.24 ± 0.24	−6.00 ± 1.03	16	4.40	−7.30	60.0	6.87
19	Kaempferol	52	0.469 ± 0.05	4.64 ± 0.29	2.19 ± 0.36	−8.05 ± 0.89	8	5.90	−8.88	45.0	7.45
20	*p*-Coumaric acid	24	0.493 ± 0.09	3.67 ± 0.16	1.80 ± 0.29	−5.04 ± 0.74	18	3.69	−6.03	43.3	6.02
Ref.	AHA	—	0.919 ± 0.02	5.08 ± 0.15	4.67 ± 0.07	−4.25 ± 0.26	—	3.11	−4.25	3/3 ^a^	5.00

Values for the 20 phytochemicals calculated across 60 retained poses per compound, corresponding to 20 independent docking runs against each of three urease receptors (PDB IDs 1E9Y, 4H9M, and 4UBP). CNNscore is the GNINA pose-quality score, whereas CNNaffinity is the neural-network-derived affinity estimate. CNN_VS was calculated for each pose as the product of CNNscore and CNNaffinity [30] and subsequently averaged across the 60 retained poses; compounds are ranked according to mean CNN_VS. ΔG is the mean Vina-derived binding energy prediction, and ΔG_best is the most favorable Vina-derived binding energy estimate observed among the retained poses. pK_calc was calculated from the Vina-derived ΔG according to Equation (4), using R = 0.0019872036 kcal·mol^−1^·K^−1^ and T = 298 K. Values are reported as the mean ± standard deviation (SD) of the 60 retained poses (20 runs × 3 receptors) set. The SD represents pose-to-pose variability and serves as an assessment of docking score stability; these values do not represent independent experimental replicates. Receptor-specific results are provided in Supplementary Table S3. Ni-coordinating poses (%) represents the percentage of poses containing at least one ligand donor atom within 3.5 Å of either catalytic Ni^2+^ ion. Contacts/pose represents the mean number of protein–ligand interactions detected per retained pose using PLIP v2.3.0. AHA, acetohydroxamic acid, is reported separately as the reference urease inhibitor. For AHA, Ni^2+^ coordination in the three receptor-validation poses is reported as 3/3. S1 ID refers to the compound numbering used in Supplementary Table S1.

**Table 8:** Cross-target Spearman rank correlations of CNN_VS scores and Vina-derived binding energy across the 20 phytochemicals.

Target Pair	CNN_VS, ρ (*p*)	Vina-Derived ΔG, ρ (*p*)
1E9Y vs. 4H9M	0.347 (*p* = 0.134)	0.910 (*p* < 0.001)
1E9Y vs. 4UBP	0.783 (*p* < 0.001)	0.901 (*p* < 0.001)
4H9M vs. 4UBP	0.570 (*p* = 0.009)	0.916 (*p* < 0.001)

Spearman’s rank correlation coefficients (ρ) calculated across the 20 phytochemicals (*n* = 20) using the compound-level CNN_VS scores and Vina-derived binding energy predictions obtained for each receptor. Reported *p*-values are two-sided. 1E9Y, *Helicobacter pylori* urease; 4H9M, jack bean (*Canavalia ensiformis*) urease; 4UBP, *Sporosarcina pasteurii* urease. Statistical significance was set at *p* < 0.05.

**Table 9:** Docking scores and active-site interaction profiles of the four highest-ranked phytochemicals according to CNN_VS and hesperidin, across the three urease receptors.

Compound	Receptor	ΔG (kcal·mol^−1^)	CNN Score	CNN Affinity	Ni^2+^ Coordinated	Closest Ni^2+^ Distance (Å)	H-Bonds	Hydrophobic Contacts	Total Contacts
Epigallocatechin	1E9Y	−7.45	0.811	5.23	2	2.33	3	3	9
	4H9M	−8.04	0.715	4.78	2	2.71	6	1	9
	4UBP	−7.87	0.688	5.33	0	—	5	1	7
Epicatechin gallate	1E9Y	−9.00	0.608	5.77	1	2.10	3	2	6
	4H9M	−9.76	0.457	5.30	2	2.10	4	2	12
	4UBP	−8.86	0.820	5.80	2	1.91	2	1	8
Gallic acid	1E9Y	−6.73	0.891	4.22	2	2.03	2	1	7
	4H9M	−6.37	0.768	4.07	2	2.64	2	1	7
	4UBP	−6.02	0.814	4.26	2	2.17	2	1	7
Epigallocatechin gallate	1E9Y	−9.19	0.759	5.72	2	2.30	5	1	8
	4H9M	−9.83	0.401	5.19	2	2.12	5	1	12
	4UBP	−8.92	0.728	5.55	2	1.79	3	2	10
Hesperidin	1E9Y	−10.38	0.338	5.46	2	2.27	6	1	10
	4H9M	−10.12	0.325	5.48	1	3.00	5	2	10
	4UBP	−10.35	0.541	6.09	1	3.26	5	1	10

The four highest-ranked phytochemicals according to mean CNN_VS (epigallocatechin, epicatechin gallate, gallic acid, and epigallocatechin gallate) and hesperidin, which exhibited the most favorable mean Vina-derived binding energy, were selected for detailed interaction analysis. For each ligand–receptor pair, values correspond to the retained pose with the most favorable (most negative) Vina-derived binding energy among 20 independent docking runs. ΔG, Vina-derived binding energy; CNNscore, GNINA pose-quality score; CNNaffinity, neural-network-derived affinity estimate. “Ni^2+^ coordinated” indicates the number of catalytic Ni^2+^ ions contacted by at least one ligand donor atom within the 3.5 Å cutoff. “Closest Ni^2+^ distance” is the shortest ligand donor atom–Ni^2+^ distance in the selected pose; — indicates that no Ni^2+^ contact satisfying the cutoff was detected. Hydrogen bonds, hydrophobic contacts, and total protein–ligand contacts were identified using PLIP v2.3.0. Total contacts include all interaction classes considered in the analysis (hydrogen bonds, metal coordination, hydrophobic contacts, salt bridges, π stacking, and π–cation interactions).

**Table 10:** Detailed active-site contacts of the selected phytochemicals in representative docking poses across the three urease receptors.

Compound	Receptor	Hydrogen Bonds	Hydrophobic	Ni^2+^/Metal	Electrostatic/π
Epigallocatechin	1E9Y	Gly279 (1.90); Arg338 (2.14); Asn168 (2.31)	Asn168 (3.51); Ala169 (3.87); Ala365 (3.90)	NI3001 (2.33); NI3002 (2.74)	His221 (5.41)
	4H9M	Arg609 (2.01); Ala636 (2.21); Arg439 (2.27); Asp633 (2.77); Gly550 (2.88); His593 (2.94)	Ala636 (3.50)	NI901 (2.71); NI902 (2.81)	-
	4UBP	Gly280 (2.07); Arg339 (2.12); Asp224 (2.72); Glu166 (2.99); Glu223 (3.0C4)	Ala170 (3.90)	-	His222 (5.46)
Epicatechin gallate	1E9Y	Asp362 (2.25); Gly279 (2.90); Asp223 (3.07)	Ala169 (3.57); Met366 (3.77)	NI3001 (2.10)	-
	4H9M	His492 (1.93); Asp494 (1.96); Ala636 (2.33); Arg439 (2.83)	Met637 (3.71); His593 (3.98)	NI902 (2.10); NI901 (3.38)	His593 (3.85); His593 (4.66); Arg609 (5.38); Arg439 (5.46)
	4UBP	His222 (1.81)	Met367 (3.44)	NI798 (1.91); NI799 (3.21)	His323 (4.06); His323 (4.93); Arg339 (5.46)
Gallic acid	1E9Y	Ala365 (1.86); Asp223 (3.35)	Met366 (3.89)	NI3002 (2.03); NI3001 (2.26)	His322 (4.63); Arg338 (5.18)
	4H9M	His492 (1.90); Ala636 (2.99)	Met637 (3.62)	NI902 (2.64); NI901 (3.34)	His593 (4.48); Arg609 (5.38)
	4UBP	His222 (1.85); Ala366 (2.57)	Met367 (3.21)	NI798 (2.17); NI799 (2.82)	His323 (4.69); Arg339 (4.77)
Epigallocatechin gallate	1E9Y	Arg338 (2.06); His322 (2.19); Met366 (2.24); His221 (2.32); Asp165 (2.69)	Asn168 (3.59)	NI3001 (2.30); NI3002 (3.19)	-
	4H9M	His492 (1.95); Asp494 (2.12); Ala636 (2.22); Arg439 (3.13); His593 (3.52)	Met637 (3.73)	NI902 (2.12); NI901 (3.38)	His593 (4.16); His593 (4.67); Arg609 (5.43); Arg439 (5.60)
	4UBP	His222 (1.87); Ala366 (2.19); His323 (3.55)	Met367 (3.38); Ala170 (3.89)	NI798 (1.79); NI799 (3.30)	His323 (4.09); His323 (5.08); Arg339 (5.43)
Hesperidin	1E9Y	Arg338 (1.99); Ala365 (2.10); Asn168 (3.03); Gly367 (3.27); Gln364 (3.29); Met366 (3.38)	Ala365 (3.92)	NI3001 (2.27); NI3002 (3.01)	His322 (4.58)
	4H9M	His492 (2.04); Arg439 (2.17); Arg609 (2.21); Ala440 (2.35); Asp494 (3.39)	Trp495 (3.63); Met637 (3.67)	NI902 (3.00)	His593 (5.14); Arg609 (5.23)
	4UBP	Ala366 (1.87); Ala170 (1.96); Gly368 (2.83); Asp224 (3.22); Leu365 (3.24)	Leu319 (3.93)	NI798 (3.26)	His323 (3.98); Arg339 (4.20); His249 (5.21)

**Table 11:** Summary of SwissADME-predicted physicochemical and drug-likeness properties of the quantified phytochemicals grouped by chemical class.

Phytochemical Class	Analyte IDs	MW (Da)	Consensus Log P	TPSA (Å^2^)	Lipinski Violations ^a^	Bioavailability Score ^b^
Phenolic acids	1, 4, 6, 17, 24	154.12–192.17	−2.32–1.38	57.53–118.22 ^c^	0–1	0.56
Phenolic aldehydes	10, 20	138.12–182.17	0.91–1.22	55.76–57.53	0	0.55
Catechins	5, 12, 22	306.27–458.37	0.93–2.20	130.61–197.37	1–2	0.17–0.55
Flavonol aglycones	47, 52	286.24–302.24	1.99–2.28	111.13–131.36	0	0.55
Flavones	50, 53, 56	270.24–284.26	2.58–2.88	79.90–90.90	0	0.55
Flavanones	48, 49	272.25–302.28	2.19	86.99–96.22	0	0.55
Flavonoid glycosides	31, 34, 35	464.38–610.56	−1.48 to −0.45	210.51–234.29	2–3	0.11–0.17

Values representing the range observed within each phytochemical class. MW, molecular weight; Consensus Log P, SwissADME consensus octanol/water partition coefficient; TPSA, topological polar surface area. Analyte IDs correspond to those used in Table 3 and Supplementary Table S4. ^a^ Number of Lipinski Rule-of-Five violations predicted by SwissADME. ^b^ SwissADME bioavailability score, representing the estimated probability of achieving at least 10% oral bioavailability. ^c^ Quinic acid showed the highest TPSA among the phenolic acids and was predicted to have low gastrointestinal absorption despite its relatively low molecular weight. Tannic acid was excluded from both molecular docking and SwissADME analyses due to its high molecular weight and structural complexity, which fall outside the small-molecule domain that was considered in the present analysis. Complete compound-level physicochemical and pharmacokinetic predictions are provided in Supplementary Table S4.

## Data Availability

The original contributions presented in this study are included in the article; further inquiries can be directed to the corresponding authors.

## Supplementary Materials

The following supporting information can be downloaded at https://www.mdpi.com/article/10.3390/molecules31203702/s1, Table S1: Full LC-MS/MS analytical method validation parameters for all 53 targeted phytochemicals with three internal standards, Table S2: Chou–Talalay median-effect analysis of ME–MEW (1:1) combination against urease inhibition across effect levels (Fa). Table S3: per-receptor molecular docking scores. Table S4: summarizes the predicted physicochemical properties and ADME profiles of the quantified compounds as assessed by SwissADME.

## Abbreviations

The following abbreviations are used in the manuscript:

PE	Petroleum-Ether
DCM	Dichloromethane
ME	Methanolic
MEW	Hydromethanolic
AQ	Aqueous
TPC	Total phenolic content
TFC	Total flavonoid content
CTC	Condensed tannin content
LOD	Limit of detection
LOQ	Limit of quantification
CNN	Convolutional Neural Network
DRI	Dose-reduction index
Fa	Fraction affected
CI	Combination index
ATCC	American type culture collection
C	Concentration
95%CI	95% Confidence interval
TPSA	Topological polar surface area
MW	Molecular weight
5PL	Five-parameter logistic
RMSD	Root-mean-square deviation
