# Supplementary material for: Targeted LC–MS/MS Phytochemical Profiling and Synergistic Anti-Urease Activity of Limoniastrum guyonianum Extracts Supported by Molecular Docking and In Silico ADME and Drug-Likeness Assessment
Source: Molecules. 2026 Oct 15;31(20):3702. doi: 10.3390/molecules31203702 (PMC13707910; doi:10.3390/molecules31203702)
Supplement: Supplementary file 1 [file molecules-31-03702-s001.zip › molecules-4591016-supplementary.pdf]

Supplementary

Supplementary Materials: Targeted LC–MS/MS Phytochemical Profiling and Synergistic Anti-Urease Activity of *Limoniastrum guyonianum* Extracts Supported by Molecular Docking and *In Silico* ADME and Drug-Likeness Assessment

1. Supplementary tables

1.1. Supplementary Table S1

Table S1 details the complete LC-MS/MS method validation parameters for all 56 targeted phytochemicals, including retention times, precursor/product ion transitions, calibration equations, linearity ranges, precision (interday/intraday RSD%), limits of detection and quantification, internal-standard-normalized response factors, and relative expanded uncertainties (U).

1.2. Supplementary Table S2

Table S2 details the Analysis not evaluable at high effect levels due to dose extrapolation beyond the experimental range CI < 1, = 1, and > 1 indicate synergism, additive, and antagonistic effects, respectively. DRI: Dose Reduction Index; ME: methanolic extract; MEW: hydromethanolic (5:1) extract. Calculations performed using CompuSyn software based on the Chou-Talalay median-effect principle.

1.2. Supplementary Table S3

Table S3 details comprehensive molecular docking results for the twenty most relevant natural compounds. Sub-tables A, B and C list receptor-resolved CNNscore and CNNaffinity metrics and predicted binding energies (mean and best poses) across three target structures (PDB IDs: 1E9Y, 4H9M, 4UBP, respectively).

1.2. Supplementary Table S4

Table S4 summarizes the predicted physicochemical properties and ADMET profiles of the quantified compounds as assessed by SwissADME, covering molecular weight, lipophilicity (XLogP), topological polar surface area (TPSA), hydrogen-bond donors/acceptors, rotatable bonds, Lipinski rule violations, blood-brain barrier penetration potential, and oral bioavailability scores.

2. Mass spectrometer and chromatograph conditions

A Shimadzu-Nexera model ultrahigh performance liquid chromatograph (UHPLC) coupled with a tandem mass spectrometer was used to accomplish quantiative evaluation of 53 phytochemicals. The reversed-phase UHPLC was equipped with an autosampler (SIL-30AC model), a column oven (CTO-10ASvp model), binary pumps (LC-30AD model), and a degasser (DGU- 20A3R model). The chromatographic conditions were optimized in order to achive optimum separation for 53 phytochemicals and overcome the suppression effects. Different columns such as Agilent Poroshell 120 EC-C18 model (150 mm×2.1 mm, 2.7 μm) and RP-C18 Inertsil ODS-4 (100 mm×2,1 mm, 2μm), different mobile phases (B) such as acetonitrile and methanol, different mobile phase additives such as ammonium formate, formic acid, ammonium acetate, and acetic acid, different column temperatures such as 25°C, 30°C, 35°C and 40°C were tried and applied until the optimum

conditions were achieved. Consequently, the chromatographic seperation was performed on a reversed phase Agilent Poroshell 120 EC-C18 model (150 mm×2.1 mm, 2.7 μm) analytical column. The column temperature was set to 40°C. The elution gradient was composed of eluent A (water+5 mM ammonium formate+0.1% formic acid) and eluent B (methanol+5 mM ammonium formate+0.1% formic acid). The following gradient elution profile was used: 20-100% B (0-25 min), 100% B (25-35 min), 20% B (35-45 min). Furthermore, the solvent flow rate and injection volume were settled as 0.5 mL/min and 5 μL, respectively.

The mass spectrometric detection was carried out using a Shimadzu LCMS-8040 model tandem mass spectrometer equipped with an electrospray ionization (ESI) source operating in both negative and positive ionization modes. LC-ESI-MS/MS data were acquired and processed by LabSolutions software (Shimadzu). The MRM (multiple reaction monitoring) mode was used for the quantification of the phytochemicals. The MRM metot was optimized to selectively detect and quantify phytochemical compounds based on the screening of specified precursor phytochemical-to-fragment ion transitions. The collision energies (CE) were optimized in order to generate optimal phtochemical fragmentation and maximal transmission of the desired product ions. The MS operatinging conditions were applied as: drying gas (N2) flow, 15 L/min; nebulizing gas (N2) flow, 3 L/min; DL temperature, 250°C; heat block temperature, 400°C, and interface temperature, 350°C.

Table S1. Analyte-specific performance characteristics of the previously validated LC–MS/MS method applied in the present study.

No	Analytes	RT[a]	M.I. (m/z)[b]	F.I. (m/z)[c]	Ion. mode	Equation	r^{2d}	Interday *RSD%*[e]	Intraday	Linearity Range (mg/L)	*LOD/LOQ* (µg/L)[f]	Recovery Interday (%)	Recovery Intraday (%)	*U*[g]	Gr. No[i]
1	Quinic acid	3.0	190.8	93.0	Neg	*y*=-0.0129989+2.97989×	0.996	0.69	0.51	0.1-5	25.7/33.3	1.0011	1.0083	0.0372	1
2	Fumaric acid	3.9	115.2	40.9	Neg	*y*=-0.0817862+1.03467×	0.995	1.05	1.02	1-50	135.7/167.9	0.9963	1.0016	0.0091	1
3	Aconitic acid	4.0	172.8	129.0	Neg	*y*=-0.7014530+32.9994×	0.971	2.07	0.93	0.1-5	16.4/31.4	0.9968	1.0068	0.0247	1
4	Gallic acid	4.4	168.8	79.0	Neg	*y*=0.0547697+20.8152×	0.999	1.60	0.81	0.1-5	13.2/17.0	1.0010	0.9947	0.0112	1
5	Epigallocatechin	6.7	304.8	219.0	Neg	*y*=-0.00494986+0.0483704×	0.998	1.22	0.73	1-50	237.5/265.9	0.9969	1.0040	0.0184	3
6	Protocatechuic acid	6.8	152.8	108.0	Neg	*y*=0.211373+12.8622×	0.957	1.43	0.76	0.1-5	21.9/38.6	0.9972	1.0055	0.0350	1
7	Catechin	7.4	288.8	203.1	Neg	*y*=-0.00370053+0.431369×	0.999	2.14	1.08	0.2-10	55.0/78.0	1.0024	1.0045	0.0221	3
8	Gentisic acid	8.3	152.8	109.0	Neg	*y*=-0.0238983+12.1494×	0.997	1.81	1.22	0.1-5	18.5/28.2	0.9963	1.0077	0.0167	1
9	Chlorogenic acid	8.4	353.0	85.0	Neg	*y*=0.289983+36.3926×	0.995	2.15	1.52	0.1-5	13.1/17.6	1.0000	1.0023	0.0213	1
10	Protocatechuic aldehyde	8.5	137.2	92.0	Neg	*y*=0.257085+25.4657×	0.996	2.08	0.57	0.1-5	15.4/22.2	1.0002	0.9988	0.0396	1
11	Tannic acid	9.2	182.8	78.0	Neg	*y*=0.0126307+26.9263×	0.999	2.40	1.16	0.05-2.5	15.3/22.7	0.9970	0.9950	0.0190	1
12	Epigallocatechin gallate	9.4	457.0	305.1	Neg	*y*=-0.0380744+1.61233×	0.999	1.30	0.63	0.2-10	61.0/86.0	0.9981	1.0079	0.0147	3
13	1,5-dicaffeoylquinic acid	9.8	515.0	191.0	Neg	*y*=-0.0164044+16.6535×	0.999	2.42	1.48	0.1-5	5.8/9.4	0.9983	0.9997	0.0306	1
14	4-OH Benzoic acid	10.5	137,2	65.0	Neg	*y*=-0.0240747+5.06492×	0.999	1.24	0.97	0.2-10	68.4/88.1	1.0032	1.0068	0.0237	1
15	Epicatechin	11.6	289.0	203.0	Neg	*y*=-0.0172078+0.0833424×	0.996	1.47	0.62	1-50	139.6/161.6	1.0013	1.0012	0.0221	3
16	Vanillic acid	11.8	166.8	108.0	Neg	*y*=-0.0480183+0.779564×	0.999	1.92	0.76	1-50	141.9/164.9	1.0022	0.9998	0.0145	1
17	Caffeic acid	12.1	179.0	134.0	Neg	*y*=0.120319+95.4610×	0.999	1.11	1.25	0.05-2.5	7.7/9.5	1.0015	1.0042	0.0152	1
18	Syringic acid	12.6	196.8	166.9	Neg	*y*=-0.0458599+0.663948×	0.998	1.18	1.09	1-50	82.3/104.5	1.0006	1.0072	0.0129	1
19	Vanillin	13.9	153.1	125.0	Pos	*y*=0.00185898+20.7382×	0.996	1.10	0.85	0.1-5	24.5/30.4	1.0009	0.9967	0.0122	1
20	Syringic aldehyde	14.6	181.0	151.1	Neg	*y*=-0.0128684+7.90153×	0.999	2.51	0.77	0.4-20	19.7/28.0	1.0001	0.9964	0.0215	1
21	Daidzin	15.2	417.1	199.0	Pos	*y*=9.45747+152.338×	0.996	2.25	1.32	0.05-2.5	7.0/9.5	0.9955	1.0017	0.0202	2
22	Epicatechin gallate	15.5	441.0	289.0	Neg	*y*=-0.0142216+1.06768×	0.997	1.63	1.28	0.1-5	19.5/28.5	0.9984	0.9946	0.0229	3
23	Piceid	17.2	391.0	135/106.9	Pos	*y*=0.00772525+25.4181×	0.999	1.94	1.16	0.05-2.5	13.8/17.8	1.0042	0.9979	0.0199	1
24	*p*-Coumaric acid	17.8	163.0	93.0	Neg	*y*=0.0249034+18.5180×	0.999	1.92	1.43	0.1-5	25.9/34.9	1.0049	1.0001	0.0194	1
25	Ferulic acid-D3-IS[h]	18.8	196.2	152.1	Neg	N.A.	N.A.	N.A.	N.A.	N.A.	N.A.	N.A.	N.A.	0.0170	1
26	Ferulic acid	18.8	192.8	149.0	Neg	*y*=-0.0735254+1.34476×	0.999	1.44	0.53	1-50	11.8/15.6	0.9951	0.9976	0.0181	1
27	Sinapic acid	18.9	222.8	193.0	Neg	*y*=-0.0929932+0.836324×	0.999	1.45	0.52	0.2-10	65.2/82.3	1.0031	1.0037	0.0317	1
28	Coumarin	20.9	146.9	103.1	Pos	*y*=0.0633397+136.508×	0.999	2.11	1.54	0.05-2.5	214.2/247.3	0.9950	0.9958	0.0383	1
29	Salicylic acid	21.8	137.2	65.0	Neg	*y*=0.239287+153.659×	0.999	1.48	1.18	0.05-2.5	6.0/8.3	0.9950	0.9998	0.0158	1
30	Cynaroside	23.7	447.0	284.0	Neg	*y*=0.280246+6.13360×	0.997	1.56	1.12	0.05-2.5	12.1/16.0	1.0072	1.0002	0.0366	2
31	Miquelianin	24.1	477.0	150.9	Neg	*y*=-0.00991585+5.50334×	0.999	1.31	0.95	0.1-5	10.6/14.7	0.9934	0.9965	0.0220	2
32	Rutin-D3-IS[h]	25.5	612.2	304.1	Neg	N.A.	N.A.	N.A.	N.A.	N.A.	N.A.	N.A.	N.A.	N.A.	2
33	Rutin	25.6	608.9	301.0	Neg	*y*=-0.0771907+2.89868×	0.999	1.38	1.09	0.1-5	15.7/22.7	0.9977	1.0033	0.0247	2
34	isoquercitrin	25.6	463.0	271.0	Neg	*y*=-0.111120+4.10546×	0.998	2.13	0.78	0.1-5	8.7/13.5	1.0057	0.9963	0.0220	2
35	Hesperidin	25.8	611.2	449.0	Pos	*y*=0.139055+13.2785×	0.999	1.84	1.35	0.1-5	19.0/26.0	0.9967	1.0043	0.0335	2
36	*o*-Coumaric acid	26.1	162.8	93.0	Neg	*y*=0.00837193+11.2147×	0.999	2.11	1.46	0.1-5	31.8/40.4	1.0044	0.9986	0.0147	1
37	Genistin	26.3	431.0	239.0	Neg	*y*=1.65808+7.57459×	0.991	2.01	1.28	0.1-5	14.9/21.7	1.0062	1.0047	0.0083	2
38	Rosmarinic acid	26.6	359.0	197.0	Neg	*y*=-0.0117238+8.04377×	0.999	1.24	0.86	0.1-5	16.2/21.2	1.0056	1.0002	0.0130	1
39	Ellagic acid	27.6	301.0	284.0	Neg	*y*=0.00877034+0.663741×	0.999	1.57	1.23	0.4-20	56.9/71.0	1.0005	1.0048	0.0364	1
40	Cosmosiin	28.2	431.0	269.0	Neg	*y*=-0.708662+8.62498×	0.998	1.65	1.30	0.1-5	6.3/9.2	0.9940	0.9973	0.0083	2
41	Quercitrin	29.8	447.0	301.0	Neg	*y*=-0.00153274+3.20368×	0.999	2.24	1.16	0.1-5	4.8/6.4	0.9960	0.9978	0.0268	2
42	Astragalin	30.4	447.0	255.0	Neg	*y*=0.00825333+3.51189×	0.999	2.08	1.72	0.1-5	6.6/8.2	0.9968	0.9957	0.0114	2
43	Nicotiflorin	30.6	592.9	255.0/284.0	Neg	*y*=0.00499333+2.62351×	0.999	1.48	1.23	0.05-2.5	11.9/16.7	0.9954	1.0044	0.0108	2
44	Fisetin	30.6	285.0	163.0	Neg	*y*=0.0365705+8.09472×	0.999	1.75	1.19	0.1-5	10.1/12.7	0.9980	1.0042	0.0231	3
45	Daidzein	34.0	253.0	223.0	Neg	*y*=-0.0329252+6.23004×	0.999	2.18	1.73	0.1-5	9.8/11.6	0.9926	0.9963	0.0370	3
46	Quercetin-D3-IS[h]	35.6	304.0	275.9	Neg	N.A.	N.A.	N.A.	N.A.	N.A.	N.A.	N.A.	N.A.	N.A.	3
47	Quercetin	35.7	301.0	272.9	Neg	*y*=+0.00597342+3.39417×	0.999	1.89	1.38	0.1-5	15.5/19.0	0.9967	0.9971	0.0175	3
48	Naringenin	35.9	270.9	119.0	Neg	*y*=-0.00393403+14.6424×	0.999	2.34	1.69	0.1-5	2.6/3.9	1.0062	1.0020	0.0392	3
49	Hesperetin	36.7	301.0	136.0/286.0	Neg	*y*=+0.0442350+6.07160×	0.999	2.47	2.13	0.1-5	7.1/9.1	0.9998	0.9963	0.0321	3
50	Luteolin	36.7	284.8	151.0/175.0	Neg	*y*=-0.0541723+30.7422×	0.999	1.67	1.28	0.05-2.5	2.6/4.1	0.9952	1.0029	0.0313	3
51	Genistein	36.9	269.0	135.0	Neg	*y*=-0.00507501+12.1933×	0.999	1.48	1.19	0.05-2.5	3.7/5.3	1.0069	1.0012	0.0337	3
52	Kaempferol	37.9	285.0	239.0	Neg	*y*=-0.00459557+3.13754×	0.999	1.49	1.26	0.05-2.5	10.2/15.4	0.9992	0.9990	0.0212	3
53	Apigenin	38.2	268.8	151.0/149.0	Neg	*y*=0.119018+34.8730×	0.998	1.17	0.96	0.05-2.5	1.3/2.0	0.9985	1.0003	0.0178	3
54	Amentoflavone	39.7	537.0	417.0	Neg	*y*=0.727280+33.3658×	0.992	1.35	1.12	0.05-2.5	2.8/5.1	0.9991	1.0044	0.0340	3
55	Chrysin	40.5	252.8	145.0/119.0	Neg	*y*=-0.0777300+18.8873×	0.999	1.46	1.21	0.05-2.5	1.5/2.8	0.9922	1.0050	0.0323	3
56	Acacetin	40.7	283.0	239.0	Neg	*y*=-0.559818+163.062×	0.997	1.67	1.28	0.02-1	1.5/2.5	0.9949	1.0011	0.0363	3

[a]RT: Retention time, [b]MI (*m/z):* Molecular ions of the standard analytes (m/z ratio), [c]FI (*m/z):* Fragment ions [d]r^2: Coefficient of determination, [e]*RSD*: Relative standard deviation, [f]*LOD/LOQ* (µg/L): Limit of detection/quantification, [g]*U* : relative expanded uncertainty at 95% confidence level (k = 2), [h]IS: Internal standard, [i]Gr. No: Represents grouping of internal standards, these numbers indicate which IS stands for which phenolic compound. N.A. not analyzed.

Table S2. Chou-Talalay median-effect analysis of ME:MEW (1:1) combination against urease inhibition across effect levels (Fa).

Fa (%)	Individual Dose (μg/mL)		Dose in Mixture (μg/mL)		CI	(95% CI)	DRI	
	D_{ME}	D_{MEW}	d_{total}	$d_{ME/MEW}$			*ME*	*MEW*
10	52.34	10.84	12.37	6.19	0.689	[0.455, 1.089]	8.46	1.75
20	90.00	22.16	21.85	10.92	0.614	[0.504, 0.732]	8.24	2.03
25	106.15	27.28	25.13	12.56	0.579	[0.489, 0.678]	8.45	2.17
30	124.35	32.35	28.36	14.18	0.552	[0.475, 0.650]	8.77	2.28
40	171.49	42.80	35.45	17.73	0.518	[0.453, 0.614]	9.67	2.41
50	241.87	54.43	44.66	22.33	0.503	[0.425, 0.597]	10.83	2.44
60	352.12	68.58	58.44	29.22	0.509	[0.398, 0.617]	12.05	2.35
70	533.16	88.62	82.88	41.44	0.545	[0.389, 0.698]	12.87	2.14
75	667.67	104.38	104.39	52.20	0.578	[0.394, 0.772]	12.79	2.00
80	847.56	134.87	140.80	70.40	0.605	[0.405, 0.877]	12.04	1.92

Analysis not evaluable at high effect levels due to dose extrapolation beyond the experimental range. CI < 1,= 1, and > 1 indicate synergism, additive, and antagonistic effects, respectively. DRI: Dose Reduction Index; ME: methanolic extract; MEW: hydromethanolic (5:1) extract. Calculations performed using CompuSyn software based on the Chou-Talalay median-effect principle.

Table S3A. Compound-level predicted binding metrics for receptor 1E9Y, summarized across 20 docking seeds.

Compound	CNN score Mean ± SD	CNN affinity Mean ± SD	Affinity[1] (kcal/mol) Mean ± SD	Best predicted energy[2] (kcal/mol)
Quinic acid	0.478 ± 0.026	4.167 ± 0.527	-5.704 ± 1.061	-6.434
Gallic acid	0.897 ± 0.005	4.237 ± 0.054	-6.724 ± 0.006	-6.732
Epigallocatechin	0.801 ± 0.016	5.211 ± 0.053	-7.435 ± 0.005	-7.447
Protocatechuic acid	0.756 ± 0.038	3.823 ± 0.033	-6.211 ± 0.150	-6.301
Protocatechuic aldehyde	0.845 ± 0.014	3.475 ± 0.046	-5.572 ± 0.193	-5.722
Tannic acid	—	—	—	—
Epigallocatechin gallate	0.738 ± 0.065	5.801 ± 0.117	-8.956 ± 0.195	-9.187
Caffeic acid	0.599 ± 0.064	4.111 ± 0.675	-5.025 ± 0.615	-5.563
Syringic aldehyde	0.616 ± 0.041	4.572 ± 0.114	-4.679 ± 0.208	-5.053
Epicatechin gallate	0.703 ± 0.063	5.822 ± 0.096	-8.685 ± 0.197	-8.997
p-Coumaric acid	0.392 ± 0.016	3.799 ± 0.160	-5.147 ± 0.208	-5.524
Miquelianin	0.671 ± 0.025	5.511 ± 0.414	-9.492 ± 0.264	-9.717
Isoquercitrin	0.701 ± 0.023	5.101 ± 0.048	-9.234 ± 0.126	-9.274
Hesperidin	0.427 ± 0.078	6.071 ± 0.371	-9.457 ± 0.602	-10.375
Quercetin	0.669 ± 0.017	5.138 ± 0.049	-8.518 ± 0.183	-8.619
Naringenin	0.697 ± 0.043	5.067 ± 0.043	-6.527 ± 0.096	-6.769
Hesperetin	0.595 ± 0.043	5.091 ± 0.092	-6.772 ± 0.149	-6.854
Luteolin	0.617 ± 0.021	4.670 ± 0.169	-8.242 ± 0.177	-8.477
Kaempferol	0.507 ± 0.020	4.853 ± 0.062	-8.800 ± 0.125	-8.883
Apigenin	0.666 ± 0.036	4.917 ± 0.176	-6.537 ± 0.209	-6.933
Acacetin	0.665 ± 0.050	4.929 ± 0.214	-6.829 ± 0.858	-8.321

Values are mean ± sample standard deviation (SD; n = 20 independent seed-level docking predictions), unless otherwise indicated. CNN score is the model's pose-confidence score; higher values indicate greater model confidence. CNN affinity is the model-predicted affinity score; higher values indicate stronger predicted affinity. [1]Affinity corresponds to the minimizedAffinity field and is reported in kcal/mol; more negative values indicate more favorable predicted binding. [2]Best

predicted energy is the minimum minimizedAffinity observed among the 20 seeds. Tannic acid was excluded from the docking dataset and is shown as an em dash (—).

Table S3B. Compound-level predicted binding metrics for receptor 4H9M, summarized across 20 docking seeds.

Compound	CNN score Mean ± SD	CNN affinity Mean ± SD	Affinity[1] (kcal/mol) Mean ± SD	Best predicted energy[2] (kcal/mol)
Quinic acid	0.587 ± 0.013	4.199 ± 0.072	-6.979 ± 0.331	-7.058
Gallic acid	0.796 ± 0.009	4.115 ± 0.033	-6.357 ± 0.040	-6.374
Epigallocatechin	0.730 ± 0.045	4.805 ± 0.088	-7.795 ± 0.219	-8.038
Protocatechuic acid	0.847 ± 0.009	3.564 ± 0.030	-6.214 ± 0.005	-6.223
Protocatechuic aldehyde	0.854 ± 0.006	3.367 ± 0.028	-5.508 ± 0.068	-5.588
Tannic acid	—	—	—	—
Epigallocatechin gallate	0.368 ± 0.030	5.104 ± 0.128	-9.620 ± 0.205	-9.828
Caffeic acid	0.728 ± 0.084	3.528 ± 0.315	-4.444 ± 0.490	-5.438
Syringic aldehyde	0.481 ± 0.043	3.741 ± 0.372	-4.641 ± 0.347	-5.175
Epicatechin gallate	0.414 ± 0.039	5.219 ± 0.095	-9.353 ± 0.353	-9.759
p-Coumaric acid	0.513 ± 0.030	3.621 ± 0.082	-5.843 ± 0.198	-6.031
Miquelianin	0.257 ± 0.023	4.682 ± 0.172	-9.231 ± 0.318	-9.857
Isoquercitrin	0.287 ± 0.040	4.661 ± 0.188	-9.099 ± 0.255	-9.557
Hesperidin	0.330 ± 0.038	5.216 ± 0.262	-9.615 ± 0.383	-10.119
Quercetin	0.596 ± 0.029	4.410 ± 0.059	-8.962 ± 0.201	-9.164
Naringenin	0.514 ± 0.016	4.652 ± 0.053	-7.154 ± 0.012	-7.168
Hesperetin	0.430 ± 0.062	4.578 ± 0.221	-6.929 ± 0.280	-7.340
Luteolin	0.528 ± 0.022	4.328 ± 0.087	-8.399 ± 0.405	-8.742
Kaempferol	0.423 ± 0.071	4.279 ± 0.127	-7.531 ± 0.212	-7.830
Apigenin	0.556 ± 0.063	4.294 ± 0.047	-7.107 ± 0.030	-7.146
Acacetin	0.585 ± 0.055	4.343 ± 0.042	-6.921 ± 0.037	-6.987

Values are mean ± sample standard deviation (SD; n = 20 independent seed-level docking predictions), unless otherwise indicated. CNN score is the model's pose-confidence score; higher values indicate greater model confidence. CNN affinity is the model-predicted affinity score; higher values indicate stronger predicted affinity. [1]Affinity corresponds to the minimizedAffinity field and is reported in kcal/mol; more negative values indicate more favorable predicted binding. [2]Best predicted energy is the minimum minimizedAffinity observed among the 20 seeds. Tannic acid was excluded from the docking dataset and is shown as an em dash (—).

Table S3C. Compound-level predicted binding metrics for receptor 4UBP, summarized across 20 docking seeds.

Compound	CNN score Mean ± SD	CNN affinity Mean ± SD	Affinity[1] (kcal/mol) Mean ± SD	Best predicted energy[2] (kcal/mol)
Quinic acid	0.591 ± 0.022	3.819 ± 0.161	-5.318 ± 0.679	-7.305
Gallic acid	0.811 ± 0.006	4.285 ± 0.027	-6.013 ± 0.008	-6.024
Epigallocatechin	0.715 ± 0.029	5.211 ± 0.107	-7.413 ± 0.432	-7.873
Protocatechuic acid	0.807 ± 0.045	3.844 ± 0.068	-5.957 ± 0.188	-6.157
Protocatechuic aldehyde	0.806 ± 0.009	3.548 ± 0.033	-5.260 ± 0.017	-5.286
Tannic acid	—	—	—	—
Epigallocatechin gallate	0.741 ± 0.027	5.601 ± 0.048	-8.677 ± 0.234	-8.924
Caffeic acid	0.576 ± 0.018	3.296 ± 0.023	-4.323 ± 0.014	-4.345
Syringic aldehyde	0.652 ± 0.055	4.011 ± 0.103	-5.257 ± 0.214	-5.519
Epicatechin gallate	0.789 ± 0.033	5.780 ± 0.055	-8.628 ± 0.144	-8.860

p-Coumaric acid	0.575 ± 0.088	3.578 ± 0.149	-4.130 ± 0.196	-4.522
Miquelianin	0.445 ± 0.054	5.218 ± 0.062	-7.770 ± 0.720	-8.933
Isoquercitrin	0.593 ± 0.068	5.350 ± 0.185	-7.751 ± 0.381	-8.507
Hesperidin	0.484 ± 0.042	6.138 ± 0.198	-9.777 ± 0.262	-10.351
Quercetin	0.626 ± 0.018	4.883 ± 0.040	-8.973 ± 0.379	-9.090
Naringenin	0.607 ± 0.043	5.061 ± 0.061	-7.242 ± 0.321	-7.563
Hesperetin	0.554 ± 0.028	5.148 ± 0.070	-7.462 ± 0.285	-7.827
Luteolin	0.600 ± 0.013	4.724 ± 0.164	-8.068 ± 0.519	-8.435
Kaempferol	0.477 ± 0.025	4.801 ± 0.146	-7.808 ± 1.212	-8.583
Apigenin	0.641 ± 0.046	4.796 ± 0.091	-7.014 ± 0.701	-7.585
Acacetin	0.682 ± 0.035	4.941 ± 0.051	-7.225 ± 0.132	-7.420

Values are mean ± sample standard deviation (SD; n = 20 independent seed-level docking predictions), unless otherwise indicated. CNN score is the model's pose-confidence score; higher values indicate greater model confidence. CNN affinity is the model-predicted affinity score; higher values indicate stronger predicted affinity. [1]Affinity corresponds to the minimizedAffinity field and is reported in kcal/mol; more negative values indicate more favorable predicted binding. [2]Best predicted energy is the minimum minimizedAffinity observed among the 20 seeds. Tannic acid was excluded from the docking dataset and is shown as an em dash (—).

Table S4. Physicochemical and predicted ADME properties for quantified compounds using SwissADME v1.5.

Compound	MW (Da)	TPSA (Å²)	Consensus Log P	HBD	HBA	nRotB	RoF Viol.	BBB	P-gp Subst.	Bioavail. Score
Quinic acid	192.17	118.22	-2.94	5	6	1	0	No	Yes	0.56
Gallic acid	170.12	97.99	0.50	4	5	1	0	No	Yes	0.56
Epigallocatechin	306.27	130.61	0.93	6	7	1	1	No	Yes	0.55
Protocatechuic acid	154.12	77.76	0.80	3	4	1	0	No	Yes	0.56
Protocatechuic aldehyde	138.12	57.53	0.65	2	3	1	0	No	Yes	0.55
Tannic acid	-	-	-	-	-	-	-	-	-	-[a]
Epigallocatechin gallate	458.37	197.37	1.91	8	11	4	2	No	Yes	0.17
Caffeic acid	180.16	77.76	1.09	3	4	2	0	No	Yes	0.56
Syringic aldehyde	182.17	55.76	0.80	1	4	3	0	No	Yes	0.55
Epicatechin gallate	442.37	177.14	2.20	7	10	4	1	No	Yes	0.17
p-Coumaric acid	164.16	57.53	1.38	2	3	2	0	No	Yes	0.56
Miquelianin	478.36	227.59	-0.45	8	13	4	2	No	Yes	0.11
Isoquercitrin	464.38	210.51	-0.54	8	12	4	2	No	Yes	0.17
Hesperidin	610.56	234.29	-1.48	8	15	7	3	No	Yes	0.17
Quercetin	302.24	131.36	1.99	5	7	1	0	No	Yes	0.55
Naringenin	272.25	86.99	2.19	3	5	1	0	No	Yes	0.55
Hesperetin	302.28	96.22	2.19	3	6	2	0	No	Yes	0.55
Luteolin	286.24	111.13	2.28	4	6	1	0	No	Yes	0.55
Kaempferol	286.24	111.13	2.28	4	6	1	0	No	Yes	0.55
Apigenin	270.24	90.90	2.58	3	5	1	0	No	Yes	0.55
Acacetin	284.26	79.90	2.88	2	5	2	0	No	Yes	0.55

[a] Tannic acid excluded from ADME profiling due to molecular weight (1701 Da) and conformational complexity exceeding SwissADME's validated predictive range for small-molecule pharmacokinetic modeling. All Consensus Log P values standardized across algorithms (XLOGP3, WLOGP, MLOGP, SILICOS-IT).
